# Effects of Replacing Fish Meal With Plant Protein Sources in Diets With or Without Jack Mackerel Meal Inclusion on Growth Performance of Red Sea Bream (*Pagrus major*)

**DOI:** 10.1155/anu/2260317

**Published:** 2026-01-02

**Authors:** Yu Jin Sim, Sung Hwoan Cho, Tae Woong Kwon, Hae Chan Shin, Hong Min Na, Yong Woo Kwon, Seong Woo Shin, Sang Hyun Lee, Ki Wook Lee, Jin Choi

**Affiliations:** ^1^ Department of Convergence Interdisciplinary Education of Maritime and Ocean Contents, National Korea Maritime and Ocean University, Busan, 49112, Republic of Korea, kmou.ac.kr; ^2^ Division of Convergence on Marine Science, National Korea Maritime and Ocean University, Busan, 49112, Republic of Korea, kmou.ac.kr; ^3^ Ocean Science and Technology School, National Korea Maritime and Ocean University, Busan, 49112, Republic of Korea, kmou.ac.kr; ^4^ Genetics and Breeding Research Center, National Institute of Fisheries Science, Geoje, 53334, Republic of Korea, nifs.go.kr; ^5^ Aquaculture Industry Research Division, East Sea Fisheries Research Institute, National Institute of Fisheries Science, Gangneung, 25435, Republic of Korea, nifs.go.kr

**Keywords:** feed enhancer, fish meal, jack mackerel meal, *Pagrus major*, plant protein source

## Abstract

Due to stagnant production and high cost of fish meal (FM), feed nutritionists are seeking reliable and affordable alternatives. However, low‐FM diets often result in poor palatability, reduced feed consumption (FC), and impaired growth. This study investigates the effects of replacing FM with plant proteins in diets with or without jack mackerel meal (JMM) inclusion on the growth performance of juvenile red sea bream (*P. major*). A three‐way {2 FM replacement sources (corn gluten meal [CGM] and soy protein concentrate [SPC]) × 2 FM replacement levels (20% and 40%) ×2 JMM inclusion (without and with)} ANOVA experimental design was applied. The control (Con) diet contained 60% FM. In the Con diet, 20% and 40% FM were replaced with CGM and SPC without or with 24% JMM inclusion, named the CGM20, CGM40, SPC20, SPC40, CGM20J, CGM40J, SPC20J, and SPC40J diets, respectively. A total of 675 juvenile fish were assigned into 27 tanks. Weight gain (WG), specific growth rate (SGR), and FC of fish fed the CGM‐replaced diets were significantly higher than those of fish fed the SPC‐replaced diets. Furthermore, dietary replacements of 20% FM achieved significantly higher WG, SGR, and FC in fish compared to those of 40% FM. Additionally, WG, SGR, and FC of fish fed the all‐plant‐protein‐replaced diets with JMM inclusion were significantly higher than those without JMM inclusion. WG, SGR, and FC of fish fed the Con diet were significantly higher than those of fish fed the CGM40, SPC20, SPC40, CGM40J, and SPC40J diets. In conclusion, up to 20% of FM can be replaced by CGM, with or without JMM inclusion, or by SPC with JMM inclusion in a 60% FM‐based diet without significantly impairing the growth performance of red sea bream.

## 1. Introduction

The world population in 2017 was 7.6 billion and is expected to reach 8.6, 9.8, and 11.2 billion in 2030, 2050, and 2100, respectively [1]. As this population growth continues along with increasing incomes, the demand for nutritious foods, especially high‐quality animal proteins, has significantly increased [2]. The products of the livestock and aquaculture industries supply the basis for protein sources in human food, contributing to well‐rounded nutritional intake for the majority of people [3]. However, livestock farming accounts for more than 50% of global soil erosion, leading to ongoing desertification [4]. Therefore, aquaculture is considered a promising industry to provide adequate protein sources to humans in the future.

In the Republic of Korea (Korea) and Japan, red sea bream (*P. major*) is one of the most valuable marine fish species. The production (6283 metric tons) of this species accounted for about 7.9% of the total (79,651 metric tons) aquaculture production in 2023 in Korea, and its value was indicated as USD 11.2 per kg of fish (USD 1 = 1350 KRW) [5], suggesting that red sea bream holds significant value. Therefore, various studies have been conducted on nutrient requirements in diets [6–11] and feeding practices [12] to improve the growth performance of red sea bream.

In general, carnivorous fish species, including red sea bream, have a high protein requirement in their diets [11, 13, 14]. In particular, dietary protein requirement for juvenile red sea bream was reported to be 52% [11]. Fish meal (FM) has been used as the main protein ingredient in fish feeds [15–17] due to its balanced amino acid (AA) profiles, high‐quality lipids including n‐3 highly unsaturated fatty acids (n‐3 HUFA), and high palatability [18]. However, the international production of FM has been reducing from 5578 metric tons in 2003 to 5059 metric tons in 2023 [19] due to overfishing [20]. In addition, the FM price has been rising from USD 640 in 2003 to USD 1815 per metric ton in 2023 [21] due to the increased requirement for FM resulting from the global extension of the aquaculture industry [22]. Thus, finding the high‐quality protein source with a stable supply and affordable price as a FM substitute is of great interest to feed nutritionists.

Diverse rendered animal proteins, such as meat meal (MM), blood meal, chicken by‐product meal and meat and bone meal, and plant proteins, such as corn gluten meal (CGM), cottonseed protein concentrate, soy protein concentrate (SPC) and soybean meal have been commonly utilized as a FM substitute in fish diets [17, 23–29]. However, the use of rendered animal proteins in animal diets is restricted due to potential safety concerns, particularly the impact of feed contamination on human bacterial disease and risk of pathogenic metabolites [30, 31]. Therefore, plant proteins have become a popular substitute for FM in fish feed because of their low cost, consistent quality, and reliable supply [17].

CGM is a by‐product left over after starch is extracted from corn [32]. It is rich in protein (over 60%), vitamins B and E, low in fiber, and free of anti‐nutritional factors (ANFs) [33, 34], indicating that CGM is a useful protein ingredient as a FM replacer in fish feeds. Several studies have reported the substitutability of FM up to 14.8% with CGM in the diets of puffer (*Takifugu fasciatus*) [29], 40% in the olive flounder (*Paralichthys olivaceus*) diet with AA (arginine [Arg], lysine [Lys], and tryptophan [Trp]) [35] and 60% in the spotted rose snapper (*Lutjanus guttatus*) diet with AA (Arg and Lys) [33], respectively, without compromising growth performance. Especially, dietary 50% FM substitution with CGM led to lower growth of red sea bream than fish fed a 60% FM‐based diet when fingerling fish were supplied with a 60% FM‐based diet or diets replacing 50% of FM by CGM, SPC, MM, and chicken by‐product meal as a single ingredient, and their combination in the 12‐week feeding study [36]. The authors stressed that the substitutability of CGM for less than 50% of FM should be assessed in the red sea bream diet.

Soybean meal accounts for more than half of all plant proteins and has high protein content but low price [37, 38]. Nevertheless, the presence of ANFs in soybean meal, such as antigenic proteins, lectins, oligosaccharides, phenolic compounds, phytates, and protease inhibitors may negatively affect nutrient digestibility and limit its use in fish feeds [37–39]. Heat treatment can deactivate some ANFs, but additional treatments are needed to reduce the concentration of other ANFs in soybeans [38]. SPC generated through soluble carbohydrate extraction from defatted soy flakes can remove or inactivate various ANFs [39]. Furthermore, SPC has a protein content of 67%–71% [40, 41] and higher nutrient digestibility compared to soybean meal in Atlantic cod (*Gadus morhua*) [42]. Previous studies have revealed the substitutability of FM up to 30% and 44% by SPC in the diets of large yellow croaker (*Larimichthys crocea*) [28] and hybrid grouper (*Epinephelus fuscoguttatus* × *E. lanceolatus*) [41], and up to 60% in the starry flounder (*Platichthys stellatus*) diet with AA (Lys and methionine [Met]) supplementation [40], respectively, without retarding growth performance. Furthermore, dietary 50% SPC substitution for FM achieved comparable growth of fingerling (initial weight [IW] of 50.2 g) red sea bream to fish fed the 60% FM‐based diet [36]. Still, the substitutability of FM by SPC in the juvenile red sea bream diet has been unknown. The size (or age) of fish can influence the substitutability of FM by the replacers in fish feeds [43, 44]. In particular, fermented soybean meal (FSM) could replace 6% and 24% FM protein in the diets of juvenile (IW of 1.2 g) and grower (IW of 148.2 g) rockfish (*Sebastes schlegeli*), respectively, without compromising growth and feed consumption (FC) when juvenile and grower rockfish were fed with a 58% FM‐based diet or diets replacing 6%, 12%, 18%, and 24% FM protein with FSM for 8 weeks [45].

High FM substitution by an alternative ingredient in fish diets generally results in poor palatability and decreased FC, ultimately leading to deteriorated growth of fish [46, 47]. Therefore, improved palatability and FC of fish can be effectively achieved by manipulation of a feed enhancer or feed ingredient having an elevating effect of FC in the low‐FM diets. In particular, AA including alanine (Ala), Arg, glycine (Gly), and proline (Pro), betaine, and inosine monophosphate (IMP) are widely recognized as the feed enhancer in fish diets [48, 49]. These nutrients are commonly rich in crude protein (CP) feed ingredient as well [50–52]. For example, IMP in the muscle extracts of jack mackerel (*Trachurus japonicus*) was an effective feed stimulant in greater amberjack (*Seriola dumerili*) [53]. Furthermore, in our previous study [54], jack mackerel meal (JMM) had the highest attractiveness to juvenile red sea bream among 18 (two animal by‐products, three crustacean meals, six FM, three mollusk meals, and four plant by‐products) protein sources, and 24% inclusion of JMM instead of FM in diets led to the greatest growth of red sea bream by improving FC. Furthermore, JMM also showed the strongest attractiveness to olive flounder [55] and rockfish [56] among 15 and 16 protein sources, respectively. In addition, JMM inclusion in diets effectively improved FC of olive flounder [57] and rockfish [58, 59], which was well reflected in their growth. JMM played as a strong feed stimulant in yellowtail (*Seriola quinqueradiata*) diets as well [60]. Therefore, JMM incorporation in developing the low‐FM diet can be a highly sustainable fish farming technique.

Regarding the issues of red sea bream aquaculture in Korea, the current study investigates the impacts of replacing various levels of FM by plant protein sources in diets with or without JMM inclusion on the growth, feed availability, blood chemistry, and chemical composition of red sea bream.

## 2. Materials and Methods

### 2.1. Arrangement of the Experimental Diets and Red Sea Bream

#### 2.1.1. Arrangement of Fish and Rearing Condition

Juvenile red sea bream used in the experiment were bought from a commercial hatchery (Tongyeong‐si, Gyeongsangnam‐do, Korea). Before starting the feeding experiment, fish were adapted to a 5‐ton circular flow‐through tank (water volume: 3.5 ton) and supplied with a commercial pellet (50% CP and 13% crude lipid [CL]) (Suhyup Feed, Uiryeong‐gun, Gyeongsangnam‐do, Korea) twice a day for 2 weeks. After the adaptation period, 675 juvenile (IW of 2.0 ± 0.01 g; mean ± SE) red sea bream were randomly divided into 27, 50‐L rectangular flow‐through tanks (water volume: 40 L) with triplicate (25 fish/tank). The mixture of underground seawater and sand‐filtered seawater at a ratio of 1:1 was provided to each tank. Water quality was checked every morning after feeding using a digital multimeter (AZ‐86031; AZ Instrument Corp., Taichung, Taiwan). The digital multimeter was calibrated weekly according to the manufacturer’s instructions. Briefly, water temperature was automatically measured by the built‐in temperature sensor of the device, and dissolved oxygen (DO) was calibrated at 100% air saturation before use. Salinity was measured in salt mode using the conductivity probe, which was rinsed with deionized water before use and soaked in KCl solution for 30 min if dehydrated. The pH electrode was calibrated using standard buffer solutions (pH 4.01, 7.01, and 10.01) before measurements. The water temperature ranged from 17.0 to 24.5°C (20.1 ± 1.83°C [mean ± SD]), DO ranged from 7.4 to 8.3 mg/L (7.8 ± 0.24 mg/L), salinity ranged from 29.8 to 31.6 g/L (30.6 ± 0.39 g/L), and pH ranged from 7.4 to 8.0 (7.6 ± 0.11). The flow rate of each tank was measured using ultrasonic flow meter (TF1100‐CH; Lanry Instruments Co., Ltd., Shanghai, China) and was 4.5 L/min. Each tank was continuously provided with adequate aeration, and the photoperiod followed natural conditions. The bottom of each tank was cleaned with siphon daily, and dead fish were promptly removed upon observation.

#### 2.1.2. Arrangement of the Experimental Diets

A three‐way [2 replacement sources (CGM and SPC) × 2 replacement levels (20% and 40%) × 2 JMM inclusion (without and with)] ANOVA experimental design was used. In the control (Con) diet, 60% FM and 13.5% defatted soybean meal, 14.5% wheat flour, and 7.0% fish oil and 2.5% soybean oil were contained as the protein, carbohydrate, and lipid sources, respectively (Table 1). Twenty percent and 40% of FM in the Con diet were substituted by CGM and SPC, respectively, named as the CGM20, CGM40, SPC20, and SPC40 diets, respectively, and then these diets were included with 24% JMM instead of FM, corresponding to 40% FM replacement level in the Con diet based on [54]’s study, in which 40% FM replacement level (24%) of JMM at the expense of FM in a 60% FM‐based diet led to the best growth performance for red sea bream. These diets were named as the CGM20J, CGM40J, SPC20J, and SPC40J diets, respectively. The nine experimental diets fulfilled the protein (CP requirement of 52%) and lipid (CL requirement of 15%) requirements of red sea bream [11], which are essential for optimal growth and metabolic function.

**Table 1:** Ingredient and chemical composition of the experimental diets (%, DM basis).

	Experimental diets								
	Con	CGM20	CGM40	SPC20	SPC40	CGM20J	CGM40J	SPC20J	SPC40J
Ingredients (%, DM)									
Fish meal (FM)^a^	60.0	48.0	36.0	48.0	36.0	24.0	12.0	24.0	12.0
Corn gluten meal (CGM)^b^	—	12.5	25.0	—	—	12.5	25.0	—	—
Soy protein concentrate (SPC)^c^	—	—	—	13.5	27.0	—	—	13.5	27.0
Jack mackerel meal (JMM)^d^	—	—	—	—	—	24.0	24.0	24.0	24.0
Defatted soybean meal	13.5	13.5	13.5	13.5	13.5	13.5	13.5	13.5	13.5
Wheat flour	14.5	13.1	11.7	12.0	9.5	13.5	12.1	12.4	9.9
Fish oil	7.0	7.9	8.8	8.0	9.0	7.5	8.4	7.6	8.6
Soybean oil	2.5	2.5	2.5	2.5	2.5	2.5	2.5	2.5	2.5
Vitamin premix^e^	1.0	1.0	1.0	1.0	1.0	1.0	1.0	1.0	1.0
Mineral premix^f^	1.0	1.0	1.0	1.0	1.0	1.0	1.0	1.0	1.0
Choline	0.5	0.5	0.5	0.5	0.5	0.5	0.5	0.5	0.5
Nutrients (%, DM)									
Dry matter	97.1	97.5	97.4	97.2	97.1	97.3	97.1	97.6	97.7
Crude protein	52.1	52.0	51.9	52.1	51.9	51.9	52.0	51.9	52.1
Crude lipid	15.1	15.0	15.3	15.3	15.1	15.3	15.2	15.2	15.2
Ash	11.2	9.7	8.3	10.3	9.5	9.5	8.0	10.1	9.3
Carbohydrate^g^	21.6	23.3	24.5	22.3	23.5	23.3	24.8	22.8	23.4

^a^Fish meal (FM) (crude protein: 72.2%, crude lipid: 8.1%, and ash: 15.1%) was imported from Chile (USD 2.44/kg FM, USD 1 = 1350 KRW).

^b^Corn gluten meal (CGM) (crude protein: 71.1%, crude lipid: 0.9%, and ash: 2.5%) was purchased from Hyunjin Livestock Distribution Co., Ltd. (Incheon Metropolitan City, Korea) (USD 0.74/kg CGM).

^c^Soy protein concentrate (SPC) (crude protein: 66.7%, crude lipid: 0.1%, and ash: 5.4%) was purchased from Solae LLC (St. Louis, MS, USA) (USD 1.33/kg SPC).

^d^Jack mackerel meal (JMM) (crude protein: 73.0%, crude lipid: 9.8%, and ash: 13.6%) was imported from Chile (USD 2.96/kg JMM).

^e^Vitamin premix (g/kg mix): L‐ascorbic acid, 121.2; DL‐*α*‐tocopheryl acetate, 18.8; thiamine hydrochloride, 2.7; riboflavin, 9.1; pyridoxine hydrochloride, 1.8; niacin, 36.4; Ca‐D‐pantothenate, 12.7; myo‐inositol, 181.8; D‐biotin, 0.27; folic acid, 0.68; p‐aminobenzoic acid, 18.2; menadione, 1.8; retinyl acetate, 0.73; cholecalciferol, 0.003; cyanocobalamin, 0.003.

^f^Mineral premix (g/kg mix): MgSO_4_^.^7H_2_O, 80.0; NaH_2_PO_4_^.^2H_2_O, 370.0; KCl, 130.0; ferric citrate, 40.0; ZnSO_4_^.^7H_2_O, 20.0; Ca‐lactate, 356.5; CuCl, 0.2; AlCl_3_^.^6H_2_O, 0.15; KI, 0.15; Na_2_Se_2_O_3_, 0.01; MnSO_4_^.^H_2_O, 2.0; CoCl_2_^.^6H_2_O, 1.0.

^g^Carbohydrate was calculated by the difference 100 − (crude protein + crude lipid + ash).

All ingredients were well blended with water at a ratio of 3:1 using a vertical mixer (B20GA; Eben Commerce Korea, Ansan‐si, Gyeonggi‐do, Korea) and pelletized using a pelletizing machine (SMC‐32; SL Company, Incheon City, Korea) with a diameter of 3 mm. The experimental diets were dried at 40°C using a machine for drying (JW‐1350ED; Jinwoo Electronics Co., Ltd., Hwaseong‐si, Gyeonggi‐do, Korea) for 24 h and kept in a freezer at –20°C until use. All experimental diets were carefully hand‐fed twice (08:00 and 17:00) a day for 8 weeks until fish were voluntarily satiated. The amount of feed supply to each tank was recorded daily after the second feeding, but uneaten diet was not collected.

### 2.2. Evaluation of the Attractiveness of the Experimental Diets to Red Sea Bream

#### 2.2.1. Arrangement of Equipment to Evaluate the Attractiveness of the Experimental Diets to Red Sea Bream

**Figure 1:**
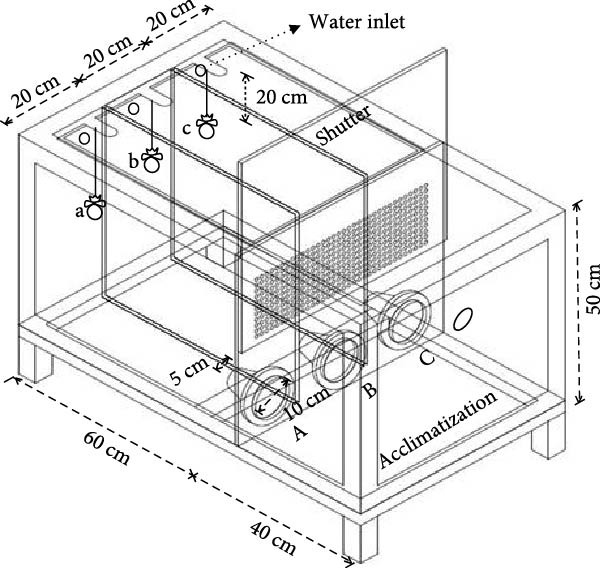
Drawing of a tank used to evaluate red sea bream’s attraction to the experimental diets (a, b, and c are the locations of different diets in each attracting chamber; A, B, and C indicate the entrances of each attracting chamber).

Three sets of reinforced flow‐through acrylic transparent tanks (100 × 60 × 50 cm; water volume: 250 L) consisted of three equally sized rectangular attracting chambers (ATCs) (60 × 20 × 50 cm each) and an acclimatization chamber (ACC) (40 × 60 × 50 cm) (Figure 1). The ATCs and ACC were separated by a vertically adjustable acrylic shutter. Each ATC had a funnel‐shaped entrance, consisting of 10 cm inlet and 5 cm outlet radii, allowing red sea bream in the ACC to freely access the experimental diets in each ATC, while preventing them from returning to the ACC. The water source was the mixture of underground seawater and sand‐filtered seawater at a ratio of 1:1. The water temperature was checked daily, and it changed from 18.5 to 19.0°C (19.0 ± 0.37°C) from the 1st to the 4th test. The flow rate of the three ATCs was maintained the same at 4.5 L/min/chamber. The same equipment used in Section 2.1.1 was employed to measure water temperature and flow rate. Each chamber was continuously supplied with adequate aeration, and the photoperiod followed natural conditions.

#### 2.2.2. Arrangement of the Experimental Red Sea Bream

Juvenile fish used to assess the attractiveness of the experimental diets were those that remained in the 5‐ton circular flow‐through tank after the start of the feeding study. Prior to the test, fish were provided with the same commercial extruded pellet used during the adaptation period, twice daily for 2 weeks. Thirty juvenile (4.7 ± 0.05 g; mean ± SE) red sea bream of similar size were randomly allotted to the ACC and adapted to the experimental conditions without diet for 48 h prior to conducting each test. Once fish were used to assess the attractiveness of the experimental diets, they were not used in any further tests.

#### 2.2.3. Evaluation of the Attractiveness of the Experimental Diets to Red Sea Bream

A knockout comparison of the experimental diets was conducted to evaluate their attractiveness to juvenile red sea bream. The experimental diets were processed into a fine powder using mechanical grinding. Then, 30 g of each diet in powdered form was wrapped in a 209 µm (mesh size) micro‐mesh screen fabric (PET 1500 32/83‐100W, Sefar, Heiden, Switzerland) and placed on the water surface of each ATC. Each test was conducted in triplicates by changing the locations of the experimental diets. The acrylic shutters were lifted to enable fish to free approach each diet in the ATC for 30 min. The raised shutter was returned to its original position in order to count the number of fish that moved toward the experimental diet in the ATCs. Movement to the ATC was recorded on video for 30 min, and the number of fish moving toward each ATC was analyzed at 10‐min intervals, calculating the attractiveness of fish for various elapsed times.

#### 2.2.4. Measurements of Growth and Biological Indices of Red Sea Bream

At the end of the 8‐week experimental trial, all live fish were fasted for a day, and then anesthetized using 50 mg/L tricaine methanesulfonate (MS‐222). The live fish in each tank were counted and weighed collectively to determine survival and weight gain (WG). Ten anesthetized fish from each tank were chosen to estimate biological indices (condition factor [CF], viscerosomatic index [VSI], and hepatosomatic index [HSI]). To calculate the growth, feed utilization, and biological indices of red sea bream, the following formulas were used: specific growth rate (SGR, %/day) = [Ln final weight of fish (g) − Ln initial weight of fish (g)] × 100/days of the feeding trial (56 days), feed efficiency (FE) = WG of fish (g)/FC of fish (g), protein efficiency ratio (PER) = WG of fish (g)/protein consumption of fish (g), protein retention (PR, %) = protein gain of fish (g) × 100/protein consumption of fish (g), CF (g/cm^3^) = body weight of fish (g) × 100/total length of fish (cm)^3^, VSI (%) = viscera weight of fish (g) × 100/body weight of fish (g), and HSI (%) = liver weight of fish (g) × 100/body weight of fish (g).

### 2.3. Plasma and Serum Parameters of Red Sea Bream

Before blood samples were taken for blood chemistry analysis, fish were individually weighed to determine CF. After the blood collection, fish were dissected to measure VSI and HSI. Blood samples were collected from the caudal veins of 5 anesthetized fish in each tank using heparinized syringes. The plasma samples were obtained by centrifuging the blood at 2700×*g* for 10 min at 4°C and were subsequently stored at –70°C. Aspartate aminotransferase (AST; catalog number: 15809542), alanine aminotransferase (ALT; catalog number: 16654035), alkaline phosphatase (ALP; catalog number: 16653964), total bilirubin (TBL; catalog number: 16654061), total cholesterol (TCO; catalog number: 16654073), triglycerides (TRG; catalog number: 16654085), total protein (TPT; catalog number: 16654097), and albumin (ALB; catalog number: 16653952) of the samples were estimated using an automatic dry‐chemistry analyzer (FUJI DRI‐CHEM NX500i; FUJIFILM Corp., Tokyo, Japan).

Blood samples were collected from the caudal vein of five anesthetized fish in each tank using syringes. The serum samples were obtained from the blood samples by centrifugation at 2700× *g* at 4°C for 10 min and stored in a deep freezer at −70°C until use. Lysozyme (LYZ) activity was determined by turbidity measurement based on Lange et al. [61]. A suspension was made by suspending 1.9 mL *M. lysodeikticus* (Sigma–Aldrich, St. Louis, MO, USA) in 0.05 M phosphate‐buffered saline at pH 6.2. A 100 µL (95 µL suspension and 5 µL serum sample) sample was incubated at 25°C for 5 min. Absorbance measurements were taken using a microplate reader (Infinite 200 PRO; Tecan Group Ltd., Männedorf, Canton of Zürich, Switzerland) at 530 nm for 60 min at 15‐min intervals. LYZ activity was estimated as the amount of enzyme required to reduce absorbance by 0.001/min.

A superoxide dismutase (SOD) analysis was performed using a SOD ELISA kit (MBS705758; MyBiosource Inc., San Diego, CA, USA) pre‐coated with a SOD‐specific antibody. The kit used a competitive inhibition enzyme immunoassay technique, showing color development in inverse proportion to the SOD concentration of the sample. The concentration was decided by constructing a standard curve. Absorbance was measured at 450 nm using the same machine as the LYZ analysis.

### 2.4. Biochemical Composition Analysis of the Experimental Diets and Whole‐Body Red Sea Bream

The main protein sources (FM, JMM, CGM, and SPC), all experimental diets, 10 juvenile red sea bream prior to the feeding experiment, and all remaining (≥13) fish in each tank were homogenized and used for the biochemical composition analysis. The chemical compositions of the samples were analyzed following the AOAC [62] standard method. The moisture content of the dry and wet samples was measured using drying oven at 105°C for 6 and 24 h, respectively. The CP and CL content were measured using the Kjeldahl method (Kjeltec 2100 Distillation Unit, Foss, Hillerød, Denmark) and the ether‐extraction method (Soxtec 2043 Fat Extraction System, Foss, Hillerød, Denmark), respectively. The ash content was evaluated using a muffle furnace for 4 h at 550°C.

The AA profiles, except for Trp, of the main protein sources, all experimental diets, and the whole‐body red sea bream were conducted using an L‐8800 automatic analyzer (Hitachi, Tokyo, Japan). Trp was analyzed using a S1125 high‐performance liquid chromatography pump system (Sykam GmbH, Eresing, Germany).

The fatty acid (FA) profiles were identified through the comparison of the samples and a known standard (37 component FAME mix CRM47885; Supelco, St. Louis, MO, USA). To conduct the FA analysis, lipids were extracted from the samples utilizing a combination of chloroform and methanol at a ratio of 2:1 (*v*/*v*), following the method by Folch et al. [63]. Subsequently, the extracted lipids were methylated with 14% BF_3_‐MeOH, and analyzed using a gas chromatograph (HP 6890; Agilent Technologies Inc., Santa Clara, CA, USA) equipped with an SP−2560 capillary column (inner diameter 100 m × 0.25 mm and film thickness 0.20 μm [Supelco, St. Louis, MO, USA]). Each FA peak was identified by comparison with the methyl ester of standard FA.

### 2.5. Statistical Analysis

All data were determined using IBM SPSS Statistics for Windows, version 24.0 (IBM Corp., Armonk, NY, USA). All means of parameters were assessed for homogeneity of variances (Levene’s test) and normality (Shapiro–Wilk) test, and no violations were observed (*p* > 0.05). Percentage data were arcsine‐transformed before significant analysis. Three‐way ANOVA was utilized to examine the effects of FM replacement source, FM replacement level, JMM inclusion, replacement source × replacement level, replacement source × JMM inclusion, replacement level × JMM inclusion, and replacement source × replacement level × JMM inclusion at a significance level of *p* < 0.05. Additionally, one‐way ANOVA was applied to compare the means of dietary treatments, including the Con diet. If significant differences (*p* < 0.05) were found among dietary treatments, post‐hoc analysis was conducted using a multiple comparison test (Tukey’s honestly significant differences test). The FA profiles of the whole‐body red sea bream were compared by principal component analysis (PCA) using SPSS version 24.0. Average linkage hierarchical clustering was performed to analyze the relationship between the FA profiles of the whole‐body fish and dietary treatments using Pearson correlation after log_2_ transformation of the data.

## 3. Results

### 3.1. The AA Profiles of the Experimental Diets

Leucine (Leu) and phenylalanine (Phe) in CGM and Arg, Phe, and Trp in SPC among essential AA (EAA) were higher than those in FM (Table 2). Histidine (His) in JMM was comparatively higher than that in FM. The content of Leu and Phe, and Arg, Phe, and Trp tended to increase with increased substitution levels of FM with CGM and SPC, respectively, in the experimental diets.

**Table 2:** Amino acid (% of the diet) profiles of the main protein sources and the experimental diets.

	Main protein sources					Experimental diets								
	FM	JMM	CGM	SPC	Requirement	Con	CGM20	CGM40	SPC20	SPC40	CGM20J	CGM40J	SPC20J	SPC40J
Essential amino acids (EAA) (%)														
Arg	4.24	4.02	2.15	4.79	2.37^a^	2.88	2.77	2.56	3.03	3.22	2.61	2.40	2.93	3.17
His	2.00	2.07	1.31	1.69	—	1.34	1.20	1.11	1.30	1.21	1.23	1.17	1.33	1.27
Ile	3.02	2.70	2.51	2.90	—	2.09	1.93	1.87	2.00	1.95	1.80	1.74	1.94	1.90
Leu	5.47	5.01	11.01	5.19	—	3.76	4.87	5.23	3.68	3.63	4.73	5.10	3.55	3.41
Lys	5.80	5.48	1.15	4.14	1.79^b^	3.84	3.03	2.84	3.66	3.48	2.90	2.76	3.52	3.40
Phe	2.93	2.62	4.09	3.40	—	2.17	2.28	2.36	2.19	2.23	2.23	2.29	2.13	2.19
Thr	3.14	2.98	2.29	2.50	—	2.19	2.03	1.85	2.07	1.97	1.99	1.81	2.00	1.97
Trp	0.54	0.53	0.30	0.64	—	0.42	0.39	0.34	0.50	0.57	0.37	0.33	0.46	0.55
Val	3.61	3.22	2.88	2.98	0.90^c^	2.54	2.40	2.36	2.50	2.38	2.33	2.25	2.37	2.29
∑EAA^d^	30.75	28.63	27.69	28.23	—	21.23	20.90	20.52	20.93	20.64	20.19	19.85	20.23	20.15
Non‐essential amino acids (NEAA) (%)														
Ala	4.59	4.42	5.94	2.79	—	2.95	3.29	3.50	2.63	2.47	3.25	3.45	2.59	2.43
Asp	6.65	6.21	4.07	7.34	—	4.73	4.58	4.26	4.94	5.00	4.51	4.20	4.73	4.88
Glu	9.29	8.70	14.10	12.20	—	7.18	8.17	8.43	7.81	8.23	8.05	8.41	7.75	8.13
Gly	4.34	4.39	1.92	2.69	—	2.89	2.61	2.42	2.78	2.47	2.65	2.44	2.79	2.51
Pro	3.00	2.97	6.58	3.52	—	2.24	2.67	2.73	2.36	2.48	2.59	2.70	2.31	2.40
Ser	2.90	2.73	3.50	3.39	—	2.11	2.19	2.33	2.15	2.25	2.15	2.25	2.13	2.19
Tyr	2.30	1.83	3.10	2.01	—	1.50	1.55	1.71	1.25	1.19	1.30	1.64	1.13	1.05
∑NEAA^e^	33.07	31.25	39.21	33.94	—	23.60	25.06	25.38	23.92	24.09	24.50	25.09	23.43	23.59

Abbreviations: Ala, alanine; Arg, arginine; Asp, aspartic acid; Glu, glutamic acid; Gly, glycine; His, histidine; Ile, isoleucine; Leu, leucine; Lys, lysine; Phe, phenylalanine; Pro, proline; Ser, serine; Thr, threonine; Trp, tryptophan; Tyr, tyrosine; Val, valine.

^a^Arginine, ^b^ lysine, and ^c^ valine requirements were obtained from Rahimnejad and Lee [9], Forster and Ogata [6], and Rahimnejad and Lee [8], respectively.

^d^∑EAA, total essential amino acids.

^e^∑NEAA, total non‐essential amino acids.

### 3.2. The FA Profiles of the Experimental Diets

CGM and SPC exhibited lower levels of total saturated FA (∑SFA), monounsaturated FA (∑MUFA), and ∑n−3 HUFA, including eicosapentaenoic acid (EPA, C20:5n−3) and docosahexaenoic acid (DHA, C22:6n−3), compared to FM (Table 3). JMM had higher ∑MUFA, but lower ∑SFA and ∑n−3 HUFA compared to FM. The ∑SFA, ∑MUFA, and ∑n−3 HUFA tended to decrease with increased substitution levels of FM with CGM and SPC in the experimental diets. The ∑SFA and ∑n−3 HUFA in all plant‐protein‐replaced diets as well as ∑MUFA, except for ∑MUFA in the CGM20J diet, were comparatively lower than those in the Con diet.

**Table 3:** Fatty acid (% of total fatty acids) profiles of the main protein sources and the experimental diets.

	Main protein sources				Experimental diets								
	FM	JMM	CGM	SPC	Con	CGM20	CGM40	SPC20	SPC40	CGM20J	CGM40J	SPC20J	SPC40J
C14:0	6.36	5.27	0.05	0.17	2.70	2.34	2.00	2.43	2.18	2.24	1.83	2.25	1.96
C16:0	21.40	19.40	12.44	23.00	14.83	14.05	13.46	14.97	15.08	13.70	12.97	14.62	14.80
C18:0	3.83	6.33	1.79	5.74	3.80	3.70	3.58	3.89	3.99	3.92	3.85	4.15	4.17
∑SFA^a^	31.59	31.00	14.28	28.91	21.33	20.09	19.04	21.29	21.25	19.86	18.65	21.02	20.93
C16:1n‐7	7.04	7.06	0.16	0.33	3.10	2.57	2.19	2.67	2.35	2.63	2.21	2.69	2.36
C17:1n‐7	1.13	1.05	0.05	0.08	0.50	0.40	0.34	0.45	0.39	0.39	0.31	0.41	0.36
C18:1n‐9	16.44	21.41	24.85	10.05	21.82	22.55	23.16	21.65	21.23	23.08	23.68	22.35	22.14
C20:1n‐9	3.49	1.50	0.31	0.10	2.69	2.49	2.40	1.81	1.68	2.18	2.13	1.63	1.42
C22:1n‐9	0.16	0.21	0.30	0.47	0.30	0.38	0.40	0.43	0.47	0.40	0.43	0.46	0.50
C24:1n‐9	3.25	3.64	0.10	0.00	1.38	1.14	0.98	1.12	0.90	1.21	1.05	1.16	1.00
∑MUFA^b^	31.51	34.87	25.77	11.03	29.79	29.53	29.47	28.13	27.02	29.89	29.76	28.70	27.83
C18:2n‐6	4.88	2.29	55.93	49.87	29.27	32.88	35.60	32.26	33.77	32.66	35.25	31.70	33.26
C18:3n‐3	0.36	0.63	2.38	6.44	3.49	3.87	4.04	3.95	4.29	3.98	4.11	4.13	4.59
C18:3n‐6	0.29	0.25	0.48	0.14	0.55	0.58	0.61	0.52	0.50	0.57	0.59	0.49	0.48
C20:4n‐6	2.04	2.51	0.00	0.11	2.02	1.88	1.74	1.94	1.90	1.93	1.85	2.00	1.98
C20:5n‐3	14.96	11.60	0.00	0.00	6.37	5.13	3.55	5.25	4.04	4.71	3.28	4.83	3.64
C22:2n‐6	0.53	0.62	0.43	0.31	0.32	0.32	0.31	0.30	0.30	0.34	0.33	0.30	0.32
C22:6n‐3	9.06	12.11	0.00	0.00	4.25	3.43	3.03	3.42	2.85	3.75	3.25	3.71	3.20
∑n–3 HUFA^c^	24.02	23.71	0.00	0.00	10.62	8.56	6.58	8.67	6.89	8.46	6.53	8.54	6.84
Unknown	4.78	4.12	0.73	3.19	2.61	2.29	2.61	2.94	4.08	2.31	2.80	3.12	3.77

^a^∑SFA, total saturated fatty acids.

^b^∑MUFA, total monounsaturated fatty acids.

^c^∑n–3 HUFA, total n–3 highly unsaturated fatty acids.

### 3.3. Attractiveness of the Experimental Diets to Fish

The attractiveness of the Con, SPC20J, and CGM20J diets to red sea bream was significantly (*p* < 0.0001 for all) stronger than that of all other diets in the 1st, 2nd, and 3rd tests, respectively, throughout the 30‐min observation period following the introduction of the experimental diets (Table 4). Lastly, in the 4th test, the attractiveness of the Con diet to fish was significantly (*p* < 0.0001) stronger than that of the SPC20J diet, but not significantly (*p* > 0.05) different from that of the CGM20J diet.

**Table 4:** Attractiveness (%) of the experimental diets to red sea bream with the elapsed time.

		Elapsed time (min)		
Test	Experimental diets	10	20	30
1st	Con	38.9 ± 2.94^a^	44.4 ± 2.94^a^	46.7 ± 3.33^a^
	SPC20	20.0 ± 1.92^b^	25.6 ± 1.11^b^	26.7 ± 1.92^b^
	CGM40J	21.1 ± 2.94^b^	24.4 ± 1.11^b^	25.6 ± 2.22^b^
	FSA^1^	20.0 ± 1.92^b^	5.6 ± 1.11^c^	1.1 ± 1.11^c^
	*p*‐Value	*p* < 0.0001	*p* < 0.0001	*p* < 0.0001
2nd	CGM20	24.4 ± 2.22^b^	30.0 ± 1.92^ab^	31.1 ± 1.11^b^
	SPC40	15.6 ± 1.11^b^	20.0 ± 1.92^bc^	21.1 ± 1.11^b^
	SPC20J	36.7 ± 1.92^a^	42.2 ± 4.01^a^	43.3 ± 3.33^a^
	FSA^1^	23.3 ± 3.33^b^	7.8 ± 2.22^c^	4.4 ± 2.22^c^
	*p*‐Value	*p* < 0.0001	*p* < 0.0001	*p* < 0.0001
3rd	CGM40	21.1 ± 2.94^b^	25.6 ± 2.94^b^	26.7 ± 1.92^b^
	CGM20J	33.3 ± 3.33^a^	38.9 ± 1.11^a^	41.1 ± 1.11^a^
	SPC40J	24.4 ± 1.11^ab^	28.9 ± 1.11^b^	30.0 ± 1.92^b^
	FSA^1^	21.1 ± 1.11^b^	6.7 ± 1.92^c^	2.2 ± 1.11^c^
	*p*‐Value	*p* < 0.0001	*p* < 0.0001	*p* < 0.0001
4th	Con	33.3 ± 1.92^a^	37.8 ± 2.94^a^	38.9 ± 2.94^a^
	CGM20J	25.6 ± 1.11^ab^	28.9 ± 1.11^ab^	31.1 ± 1.11^ab^
	SPC20J	22.2 ± 1.11^b^	25.6 ± 1.11^b^	27.8 ± 2.22^b^
	FSA^1^	18.9 ± 1.11^b^	7.8 ± 2.94^c^	2.2 ± 2.22^c^
	*p*‐Value	*p* < 0.0001	*p* < 0.0001	*p* < 0.0001

*Note:* Values (means of triplicates ± SE) in the same column sharing the same superscript letter are not significantly different (*p*  > 0.05).

^1^FSA is the percentage of fish that stayed in the acclimatization chamber after the 30‐min exposure to each diet.

### 3.4. Survival and Growth Performance of Red Sea Bream

Survival of red sea bream ranged from 94.7% to 98.7% but was not significantly affected by dietary replacement source (*p* > 0.4), replacement level (*p* > 0.1), and JMM inclusion (*p* > 0.4) (Table 5). The WG and SGR of red sea bream fed the CGM‐replaced diets (7.3 g/fish and 2.75%/day) were significantly (*p* < 0.001 for both) greater than those of fish fed the SPC‐replaced diets (6.9 g/fish and 2.65%/day, respectively). Moreover, dietary replacements of 20% FM (7.7 g/fish and 2.82%/day) attained significantly (*p* < 0.0001 for both) higher WG and SGR than those of 40% FM (6.5 g/fish and 2.58%/day, respectively). In addition, all plant‐replaced diets with JMM inclusion (7.4 g/fish and 2.76%/day) achieved significantly (*p* < 0.0001 and *p* < 0.001, respectively) higher WG and SGR than those without JMM inclusion (6.9 g/fish and 2.64%/day, respectively). However, none of their interactions (replacement source × replacement level, replacement source × JMM, replacement level × JMM, and replacement source × replacement level × JMM) significantly (*p* > 0.05 for all) influence WG and SGR. WG and SGR of red sea bream fed the Con diet were comparable to those of fish fed the CGM20, CGM20J, and SPC20J diets, but significantly (*p* < 0.0001 for both) greater than those of fish fed the all other diets in multiple comparison test.

**Table 5:** Survival (%), weight gain (WG, g/fish), and specific growth rate (SGR, %/day) of red sea bream fed the experimental diets for 8 weeks.

Experimental diets	Initial weight (g/fish)	Final weight (g/fish)	Survival (%)	WG (g/fish)	SGR^1^ (%/day)
Con	2.0 ± 0.03	10.4 ± 0.19	97.3 ± 1.33	8.4 ± 0.16^a^	2.93 ± 0.009^a^
CGM20	2.0 ± 0.03	9.7 ± 0.21	98.7 ± 1.33	7.7 ± 0.19^abc^	2.84 ± 0.026^ab^
CGM40	2.1 ± 0.03	8.5 ± 0.15	96.0 ± 2.31	6.5 ± 0.15^ef^	2.54 ± 0.043^d^
SPC20	2.0 ± 0.05	9.4 ± 0.17	97.3 ± 1.33	7.3 ± 0.15^bcd^	2.74 ± 0.042^bc^
SPC40	2.0 ± 0.03	8.0 ± 0.11	94.7 ± 2.67	6.0 ± 0.13^f^	2.45 ± 0.046^d^
CGM20J	2.0 ± 0.03	10.0 ± 0.14	98.7 ± 1.33	8.0 ± 0.14^ab^	2.90 ± 0.027^ab^
CGM40J	2.0 ± 0.03	9.1 ± 0.13	97.3 ± 1.33	7.1 ± 0.14^cde^	2.72 ± 0.036^bc^
SPC20J	2.0 ± 0.05	9.7 ± 0.18	97.3 ± 1.33	7.7 ± 0.13^abc^	2.82 ± 0.008^ab^
SPC40J	2.0 ± 0.07	8.6 ± 0.18	97.3 ± 1.33	6.6 ± 0.16^def^	2.59 ± 0.054^cd^
*p*‐Value	—	—	*p* > 0.8	*p* < 0.0001	*p* < 0.0001
Main effect: replacement source					
CGM	—	—	97.7 ± 0.95	7.3 ± 0.24^A^	2.75 ± 0.054^A^
SPC	—	—	96.7 ± 1.02	6.9 ± 0.26^B^	2.65 ± 0.056^B^
Main effect: replacement level					
20%	—	—	98.0 ± 0.74	7.7 ± 0.12^A^	2.82 ± 0.026^A^
40%	—	—	96.3 ± 1.12	6.5 ± 0.17^B^	2.58 ± 0.043^B^
Main effect: jack mackerel meal					
Without	—	—	96.7 ± 1.18	6.9 ± 0.27^B^	2.64 ± 0.060^B^
With	—	—	97.7 ± 0.73	7.4 ± 0.22^A^	2.76 ± 0.046^A^
Three‐way ANOVA					
Replacement source	—	—	*p* > 0.4	*p* < 0.001	*p* < 0.001
Replacement level	—	—	*p* > 0.1	*p* < 0.0001	*p* < 0.0001
Jack mackerel meal	—	—	*p* > 0.4	*p* < 0.0001	*p* < 0.001
Replacement source × replacement level	—	—	*p* > 0.7	*p* > 0.5	*p* > 0.7
Replacement source × Jack mackerel meal	—	—	*p* > 0.7	*p* > 0.9	*p* > 0.8
Replacement level × Jack mackerel meal	—	—	*p* > 0.4	*p* > 0.1	*p* > 0.1
Replacement source × replacement level × Jack mackerel meal	—	—	*p* > 0.7	*p* > 0.9	*p* > 0.5

*Note:* Values (means of triplicates ± SE) with different lowercase and uppercase letters indicate significant differences (*p* < 0.05) based on Tukey’s honestly significant differences test and three‐way ANOVA, respectively.

^1^Specific growth rate (SGR, %/day) = [Ln final weight of fish (g) − Ln initial weight of fish (g)] × 100/days of the feeding trial.

### 3.5. Feed Availability and Biological Indices of Red Sea Bream

The CGM‐replaced diets (7.26 g/fish) achieved significantly (*p* < 0.001) higher FC of fish than the SPC‐replaced diets (6.81 g/fish) (Table 6). Dietary replacements of 20% FM (7.57 g/fish) attained significantly (*p* < 0.0001) higher FC than those of 40% FM (6.51 g/fish). Furthermore, all plant‐replaced diets with JMM inclusion (7.27 g/fish) achieved significantly (*p* < 0.001) higher FC than those without JMM inclusion (6.81 g/fish). FC of fish fed the Con diet was significantly (*p* < 0.0001) higher than that of fish fed the CGM40, SPC20, SPC40, CGM40J, and SPC40J diets, but not significantly (*p* > 0.05) different from that of fish fed CGM20, CGM20J, and SPC20J diets.

**Table 6:** Feed consumption (FC, g/fish), feed efficiency (FE), protein efficiency ratio (PER), protein retention (PR, %), condition factor (CF, g/cm^3^), viscerosomatic index (VSI, %), and hepatosomatic index (HSI, %) of red sea bream fed the experimental diets for 8 weeks.

Experimental diets	FC (g/fish)	FE^1^	PER^2^	PR^3^ (%)	CF^4^ (g/cm^3^)	VSI^5^ (%)	HSI^6^ (%)
Con	8.07 ± 0.183^a^	1.04 ± 0.013	2.00 ± 0.026	32.17 ± 0.650	1.88 ± 0.017^a^	6.53 ± 0.024^d^	1.19 ± 0.038^c^
CGM20	7.61 ± 0.244^ab^	1.02 ± 0.025	1.96 ± 0.048	31.67 ± 0.720	1.72 ± 0.040^ab^	7.52 ± 0.228^bcd^	1.24 ± 0.012^c^
CGM40	6.40 ± 0.207^d^	1.01 ± 0.019	1.95 ± 0.037	31.30 ± 0.550	1.63 ± 0.055^b^	8.70 ± 0.215^a^	1.45 ± 0.013^a^
SPC20	7.26 ± 0.078^b^	1.01 ± 0.029	1.94 ± 0.055	31.40 ± 0.767	1.72 ± 0.044^ab^	7.55 ± 0.059^bc^	1.30 ± 0.046^bc^
SPC40	5.97 ± 0.118^d^	1.00 ± 0.005	1.93 ± 0.010	30.66 ± 0.399	1.61 ± 0.035^b^	8.99 ± 0.084^a^	1.49 ± 0.006^a^
CGM20J	7.86 ± 0.149^ab^	1.02 ± 0.011	1.97 ± 0.021	32.32 ± 0.274	1.74 ± 0.021^ab^	7.19 ± 0.342^cd^	1.21 ± 0.020^c^
CGM40J	7.19 ± 0.137^bc^	0.99 ± 0.009	1.90 ± 0.018	30.30 ± 0.044	1.63 ± 0.047^b^	8.30 ± 0.177^ab^	1.42 ± 0.026^ab^
SPC20J	7.53 ± 0.125^ab^	1.02 ± 0.004	1.97 ± 0.008	31.71 ± 0.668	1.73 ± 0.034^ab^	7.33 ± 0.073^bcd^	1.26 ± 0.029^c^
SPC40J	6.48 ± 0.036^cd^	1.02 ± 0.021	1.96 ± 0.040	31.97 ± 0.577	1.63 ± 0.037^b^	8.61 ± 0.336^a^	1.43 ± 0.023^ab^
*p*‐Value	*p* < 0.0001	*p* > 0.6	*p* > 0.6	*p* > 0.2	*p* < 0.003	*p* < 0.001	*p* < 0.001
Main effect: replacement source							
CGM	7.26 ± 0.227^A^	1.01 ± 0.010	1.95 ± 0.020	31.39 ± 0.336	1.68 ± 0.029	7.93 ± 0.257	1.33 ± 0.039^B^
SPC	6.81 ± 0.235^B^	1.01 ± 0.010	1.95 ± 0.019	31.44 ± 0.370	1.67 ± 0.028	8.12 ± 0.273	1.37 ± 0.037^A^
Main effect: replacement level							
20%	7.57 ± 0.115^A^	1.02 ± 0.011	1.96 ± 0.021	31.77 ± 0.356	1.73 ± 0.019^A^	7.40 ± 0.123^B^	1.25 ± 0.019^B^
40%	6.51 ± 0.178^B^	1.01 ± 0.009	1.94 ± 0.017	31.06 ± 0.331	1.63 ± 0.023^B^	8.65 ± 0.147^A^	1.45 ± 0.014^A^
Main effect: jack mackerel meal							
Without	6.81 ± 0.259^B^	1.01 ± 0.011	1.95 ± 0.022	31.26 ± 0.354	1.67 ± 0.030	8.19 ± 0.259^A^	1.37 ± 0.040^A^
With	7.27 ± 0.199^A^	1.01 ± 0.009	1.95 ± 0.017	31.58 ± 0.373	1.68 ± 0.026	7.86 ± 0.262^B^	1.33 ± 0.037^B^
Three‐way ANOVA							
Replacement source	*p* < 0.001	*p* > 0.7	*p* > 0.8	*p* > 0.9	*p* > 0.7	*p* > 0.2	*p* < 0.03
Replacement level	*p* < 0.0001	*p* > 0.3	*p* > 0.3	*p* > 0.08	*p* < 0.003	*p* < 0.0001	*p* < 0.0001
Jack mackerel meal	*p* < 0.001	*p* > 0.8	*p* > 0.8	*p* > 0.4	*p* > 0.6	*p* < 0.04	*p* < 0.04
Replacement source × replacement level	*p* > 0.2	*p* > 0.5	*p* > 0.5	*p* > 0.2	*p* > 0.9	*p* > 0.5	*p* > 0.4
Replacement source × Jack mackerel meal	*p* > 0.5	*p* > 0.3	*p* > 0.3	*p* > 0.2	*p* > 0.9	*p* > 0.8	*p* > 0.5
Replacement level × Jack mackerel meal	*p* > 0.08	*p* > 0.6	*p* > 0.4	*p* > 0.6	*p* > 0.9	*p* > 0.7	*p* > 0.6
Replacement source × replacement level × Jack mackerel meal	*p* > 0.4	*p* > 0.3	*p* > 0.4	*p* > 0.1	*p* > 0.9	*p* > 0.8	*p* > 0.7

*Note:* Values (means of triplicates ± SE) with different lowercase and uppercase letters indicate significant differences (*p* < 0.05) based on Tukey’s honestly significant differences test and three‐way ANOVA, respectively.

^1^Feed efficiency (FE) = [total final weight of fish (g) − total initial weight of fish (g) + total weight of dead fish (g)]/total feed consumption (g).

^2^Protein efficiency ratio (PER) = weight gain of fish (g)/protein consumption of fish (g).

^3^Protein retention (PR, %) = protein gain of fish (g) × 100/protein consumption of fish (g).

^4^Condition factor (CF, g/cm^3^) = body weight of fish (g) × 100/total length of fish (cm)^3^.

^5^Viscerosomatic index (VSI, %) = viscera weight of fish (g) × 100/body weight of fish (g).

^6^Hepatosomatic index (HSI, %) = liver weight of fish (g) × 100/body weight of fish (g).

FE of fish ranged from 0.99 to 1.04, while PER ranged from 1.90 to 2.00 and PR ranged from 30.30% to 32.32%, but these parameters were not significantly (*p* > 0.05 for all) affected by dietary replacement source, replacement level, or JMM inclusion.

Dietary replacement source and JMM inclusion did not significantly (*p* > 0.7 and *p* > 0.6, respectively) impact CF of fish. However, dietary replacements of 20% FM (1.73 g/cm^3^) attained significantly (*p* < 0.003) higher CF of fish than those of 40% FM (1.63 g/cm^3^). Dietary replacement source did not significantly (*p* > 0.2) influence VSI of fish. However, dietary replacements of 40% FM (8.65%) achieved significantly (*p* < 0.0001) higher VSI than those of 20% FM replacements (7.40%). All plant‐replaced diets without JMM inclusion (8.19%) also attained significantly (*p* < 0.04) higher VSI than those with JMM inclusion (7.86%). The SPC‐replaced diets (1.37%) attained significantly (*p* < 0.03) higher HSI than the CGM‐replaced diets (1.33%). Dietary replacements of 40% FM (1.45%) were significantly (*p* < 0.0001) higher HSI than those of 20% FM (1.25%). In addition, HSI of fish fed the all plant‐replaced diets without JMM inclusion (1.37%) was significantly (*p* < 0.04) higher than that of fish fed the all plant‐replaced diets with JMM inclusion (1.33%). According to the multiple comparison test, CF of fish fed the Con diet was significantly (*p* < 0.003) higher than that of fish fed the CGM40, SPC40, CGM40J, and SPC40J diets. VSI of fish fed the CGM40, SPC40, and SPC40J diets was also significantly (*p* < 0.001) higher than that of fish fed the Con, CGM20, SPC20, CGM20J, and SPC20J diets. In addition, HSI of fish fed the CGM40 and SPC40 diets was significantly (*p* < 0.001) higher than that of fish fed the Con, CGM20, SPC20, CGM20J, and SPC20J diets.

### 3.6. Blood Chemistry of Red Sea Bream

Plasma AST changed from 51.6 to 55.4 U/L, ALT changed from 8.2 to 9.0 U/L, ALP changed from 197.0 to 213.2 U/L, TBL changed from 0.7 to 1.1 mg/dL, TCO changed from 239.8 to 252.1 mg/dL, TRG changed from 210.4 to 220.7 mg/dL, TPT changed from 3.4 to 3.7 g/dL, and ALB changed from 0.9 to 1.1 g/dL (Table 7). In addition, serum LYZ activity varied from 80.5 to 108.8 U/mL and SOD varied from 1.7 to 2.0 ng/mL. None of blood parameters in fish were significantly (*p* > 0.05 for all) changed by dietary replacement source, replacement level, or JMM inclusion.

**Table 7:** Plasma and serum parameters of red sea bream fed the experimental diets for 8 weeks.

Experimental diets	Plasma parameters								Serum parameters	
	AST (U/L)	ALT (U/L)	ALP (U/L)	TBL (mg/dL)	TCO (mg/dL)	TRG (mg/dL)	TPT (g/dL)	ALB (g/dL)	LYZ activity (U/mL)	SOD (ng/mL)
Con	55.4 ± 2.19	8.7 ± 0.19	200.2 ± 1.42	1.0 ± 0.14	249.1 ± 4.04	213.0 ± 9.43	3.6 ± 0.04	1.0 ± 0.06	80.5 ± 12.50	1.7 ± 0.07
CGM20	53.4 ± 1.94	8.7 ± 0.19	213.2 ± 7.52	0.8 ± 0.06	245.1 ± 5.25	220.7 ± 6.16	3.5 ± 0.12	1.0 ± 0.07	85.2 ± 11.53	1.8 ± 0.12
CGM40	51.6 ± 1.44	8.6 ± 0.29	197.0 ± 5.10	1.1 ± 0.08	251.1 ± 6.49	216.7 ± 11.51	3.4 ± 0.09	1.0 ± 0.14	107.3 ± 12.05	1.7 ± 0.03
SPC20	55.4 ± 1.06	8.3 ± 0.69	198.6 ± 2.62	1.1 ± 0.13	239.8 ± 8.66	213.4 ± 3.99	3.7 ± 0.12	1.0 ± 0.21	108.8 ± 18.48	1.7 ± 0.07
SPC40	55.1 ± 2.19	8.3 ± 0.58	212.4 ± 6.78	0.9 ± 0.18	242.6 ± 8.56	217.4 ± 6.76	3.6 ± 0.17	0.9 ± 0.07	97.5 ± 18.77	1.9 ± 0.16
CGM20J	53.6 ± 2.35	8.6 ± 0.91	208.8 ± 6.56	0.9 ± 0.05	249.8 ± 2.60	211.3 ± 2.50	3.7 ± 0.22	1.0 ± 0.18	97.2 ± 16.60	1.7 ± 0.09
CGM40J	55.0 ± 1.53	8.4 ± 0.62	204.4 ± 7.06	0.8 ± 0.20	241.0 ± 3.72	210.8 ± 8.95	3.4 ± 0.19	1.1 ± 0.09	103.8 ± 19.82	1.8 ± 0.14
SPC20J	54.3 ± 2.08	9.0 ± 0.84	211.0 ± 2.96	0.7 ± 0.09	252.1 ± 8.37	210.4 ± 3.64	3.5 ± 0.13	1.1 ± 0.13	95.7 ± 11.49	2.0 ± 0.16
SPC40J	53.0 ± 2.36	8.2 ± 0.48	205.3 ± 5.59	1.0 ± 0.07	246.9 ± 5.64	215.6 ± 2.75	3.4 ± 0.19	1.0 ± 0.07	95.2 ± 12.68	1.7 ± 0.07
*p*‐Value	*p* > 0.8	*p* > 0.9	*p* > 0.3	*p* > 0.4	*p* > 0.8	*p* > 0.9	*p* > 0.8	*p* > 0.9	*p* > 0.9	*p* > 0.7
Main effect: replacement source										
CGM	53.4 ± 1.07	8.6 ± 0.30	205.9 ± 4.10	0.9 ± 0.07	246.8 ± 2.89	214.9 ± 4.45	3.5 ± 0.09	1.0 ± 0.07	98.4 ± 8.62	1.8 ± 0.06
SPC	54.5 ± 1.10	8.5 ± 0.36	206.8 ± 3.23	0.9 ± 0.08	245.3 ± 4.47	214.2 ± 2.56	3.6 ± 0.09	1.0 ± 0.07	99.3 ± 8.46	1.8 ± 0.07
Main effect: replacement level										
20%	54.2 ± 1.05	8.6 ± 0.38	207.9 ± 3.49	0.9 ± 0.07	246.7 ± 3.91	214.0 ± 2.68	3.6 ± 0.09	1.0 ± 0.08	96.7 ± 8.35	1.8 ± 0.07
40%	53.7 ± 1.14	8.4 ± 0.27	204.8 ± 3.81	0.9 ± 0.08	245.4 ± 3.62	215.1 ± 4.36	3.4 ± 0.09	1.0 ± 0.05	101.0 ± 8.65	1.8 ± 0.06
Main effect: jack mackerel meal										
Without	53.9 ± 1.06	8.5 ± 0.26	205.3 ± 4.12	1.0 ± 0.08	244.6 ± 4.15	217.1 ± 4.09	3.6 ± 0.08	1.0 ± 0.07	99.7 ± 8.86	1.8 ± 0.06
With	54.0 ± 1.14	8.6 ± 0.40	207.4 ± 3.16	0.9 ± 0.07	247.5 ± 3.27	212.0 ± 2.81	3.5 ± 0.10	1.0 ± 0.07	98.0 ± 8.20	1.8 ± 0.08
Three‐way ANOVA										
Replacement source	*p* > 0.4	*p* > 0.8	*p* > 0.8	*p* > 0.7	*p* > 0.7	*p* > 0.8	*p* > 0.9	*p* > 0.8	*p* > 0.9	*p* > 0.4
Replacement level	*p* > 0.7	*p* > 0.5	*p* > 0.4	*p* > 0.6	*p* > 0.7	*p* > 0.8	*p* > 0.1	*p* > 0.8	*p* > 0.7	*p* > 0.8
Jack mackerel meal	*p* > 0.9	*p* > 0.8	*p* > 0.6	*p* > 0.2	*p* > 0.5	*p* > 0.2	*p* > 0.5	*p* > 0.5	*p* > 0.8	*p* > 0.8
Replacement source × replacement level	*p* > 0.8	*p* > 0.7	*p* > 0.09	*p* > 0.5	*p* > 0.9	*p* > 0.4	*p* > 0.9	*p* > 0.5	*p* > 0.3	*p* > 0.4
Replacement source × Jack mackerel meal	*p* > 0.2	*p* > 0.6	*p* > 0.8	*p* > 0.6	*p* > 0.2	*p* > 0.5	*p* > 0.3	*p* > 0.5	*p* > 0.5	*p* > 0.7
Replacement level × Jack mackerel meal	*p* > 0.6	*p* > 0.6	*p* > 0.6	*p* > 0.6	*p* > 0.2	*p* > 0.8	*p* > 0.9	*p* > 0.8	*p* > 0.9	*p* > 0.5
Replacement source × replacement level × Jack mackerel meal	*p* > 0.4	*p* > 0.6	*p* > 0.07	*p* < 0.03	*p* > 0.7	*p* > 0.9	*p* > 0.6	*p* > 0.5	*p* > 0.5	*p* > 0.1

*Note:* Values (means of triplicates ± SE) are presented.

Abbreviations: ALB, albumin; ALP, alkaline phosphatase; ALT, alanine aminotransferase; AST, aspartate aminotransferase; LYZ activity, Lysozyme activity; SOD, superoxide dismutase; TBL, total bilirubin; TCO, total cholesterol; TPT, total protein; TRG, triglycerides.

### 3.7. The Chemical Composition of the Whole‐Body Red Sea Bream

The content of moisture of the whole‐body fish varied from 69.7% to 70.5%, CP varied from 16.3% to 16.6%, CL varied from 7.2% to 7.7%, and ash varied from 4.2% to 4.4% (Table 8). The chemical composition of the whole‐body fish was significantly (*p* > 0.05 for all) unaffected by dietary replacement source, replacement level, or JMM inclusion.

**Table 8:** The whole‐body chemical composition (% of wet weight) of red sea bream fed the experimental diets for 8 weeks.

Experimental diets	Moisture	Crude protein	Crude lipid	Ash
Con	69.8 ± 0.41	16.4 ± 0.10	7.3 ± 0.23	4.4 ± 0.25
CGM20	69.8 ± 0.09	16.5 ± 0.06	7.7 ± 0.28	4.3 ± 0.17
CGM40	70.1 ± 0.12	16.4 ± 0.12	7.7 ± 0.09	4.3 ± 0.18
SPC20	70.3 ± 0.18	16.5 ± 0.06	7.2 ± 0.40	4.4 ± 0.15
SPC40	70.5 ± 0.15	16.3 ± 0.09	7.4 ± 0.15	4.3 ± 0.09
CGM20J	70.1 ± 0.61	16.6 ± 0.07	7.5 ± 0.20	4.3 ± 0.20
CGM40J	70.4 ± 0.50	16.3 ± 0.12	7.4 ± 0.31	4.2 ± 0.10
SPC20J	69.9 ± 0.47	16.5 ± 0.32	7.3 ± 0.26	4.4 ± 0.29
SPC40J	69.7 ± 0.32	16.6 ± 0.44	7.4 ± 0.15	4.3 ± 0.18
*p*‐Value	*p* > 0.7	*p* > 0.9	*p* > 0.9	*p* > 0.9
Main effect: replacement source				
CGM	70.1 ± 0.22	16.5 ± 0.06	7.6 ± 0.13	4.3 ± 0.09
SPC	70.1 ± 0.20	16.5 ± 0.15	7.3 ± 0.14	4.3 ± 0.10
Main effect: replacement level		—	—	—
20%	70.1 ± 0.22	16.5 ± 0.09	7.4 ± 0.17	4.3 ± 0.11
40%	70.2 ± 0.20	16.4 ± 0.13	7.5 ± 0.11	4.3 ± 0.08
Main effect: jack mackerel meal				
Without	70.2 ± 0.12	16.4 ± 0.05	7.5 ± 0.16	4.3 ± 0.08
With	70.0 ± 0.27	16.5 ± 0.15	7.4 ± 0.12	4.3 ± 0.11
Three‐way ANOVA				
Replacement source	*p* > 0.9	*p* > 0.9	*p* > 0.2	*p* > 0.6
Replacement level	*p* > 0.6	*p* > 0.5	*p* > 0.8	*p* > 0.6
Jack mackerel meal	*p* > 0.4	*p* > 0.6	*p* > 0.6	*p* > 0.8
Replacement source × replacement level	*p* > 0.5	*p* > 0.5	*p* > 0.6	*p* > 0.8
Replacement source × Jack mackerel meal	*p* > 0.08	*p* > 0.6	*p* > 0.4	*p* > 0.7
Replacement level × Jack mackerel meal	*p* > 0.6	*p* > 0.8	*p* > 0.7	*p* > 0.9
Replacement source × replacement level × Jack mackerel meal	*p* > 0.7	*p* > 0.3	*p* > 0.8	*p* > 0.6

*Note:* Values (means of triplicates ± SE) are presented.

### 3.8. The AA Profiles of the Whole‐Body Red Sea Bream

Among EAA, Arg and Leu were significantly (*p* < 0.05, *p* < 0.002, respectively) affected by dietary replacement source, while among non‐EAA (NEAA), Ala, aspartic acid (Asp), and tyrosine (Tyr) showed significant (*p* < 0.05, *p* < 0.006, and *p* < 0.02, respectively) differences in the whole‐body fish (Table 9). Isoleucine (Ile) among EAA of the whole‐body fish was significantly (*p* < 0.05) affected by dietary replacement level. However, JMM inclusion did not significantly (*p* > 0.05 for all) impact the whole‐body AA profiles of fish. However, the AA profiles of the whole‐body fish were significantly (*p* > 0.05 for all) unaffected by dietary treatments in the multiple comparison test.

**Table 9:** The whole‐body amino acid (% of wet weight) profiles of red sea bream fed the experimental diets for 8 weeks.

Experimental diets	Essential amino acids (EAA) (%)									Non‐essential amino acids (NEAA) (%)						
	Arg	His	Ile	Leu	Lys	Phe	Thr	Trp	Val	Ala	Asp	Glu	Gly	Pro	Ser	Tyr
Con	0.95 ± 0.020	0.35 ± 0.029	0.56 ± 0.029	1.08 ± 0.026	1.24 ± 0.026	0.59 ± 0.023	0.68 ± 0.023	0.11 ± 0.012	0.67 ± 0.029	1.09 ± 0.020	1.43 ± 0.029	2.10 ± 0.026	1.24 ± 0.032	0.74 ± 0.026	0.69 ± 0.026	0.43 ± 0.020
CGM20	0.93 ± 0.026	0.33 ± 0.026	0.55 ± 0.020	1.10 ± 0.017	1.20 ± 0.032	0.62 ± 0.029	0.66 ± 0.032	0.11 ± 0.015	0.65 ± 0.026	1.12 ± 0.026	1.40 ± 0.029	2.14 ± 0.026	1.21 ± 0.017	0.76 ± 0.032	0.72 ± 0.020	0.46 ± 0.026
CGM40	0.91 ± 0.032	0.31 ± 0.029	0.53 ± 0.017	1.11 ± 0.017	1.16 ± 0.020	0.63 ± 0.032	0.62 ± 0.026	0.11 ± 0.015	0.63 ± 0.026	1.15 ± 0.032	1.39 ± 0.026	2.16 ± 0.026	1.20 ± 0.026	0.79 ± 0.023	0.73 ± 0.020	0.44 ± 0.026
SPC20	0.95 ± 0.026	0.35 ± 0.020	0.57 ± 0.035	1.07 ± 0.015	1.22 ± 0.026	0.59 ± 0.023	0.67 ± 0.017	0.12 ± 0.023	0.66 ± 0.020	1.09 ± 0.032	1.45 ± 0.032	2.13 ± 0.020	1.24 ± 0.023	0.74 ± 0.026	0.73 ± 0.026	0.40 ± 0.026
SPC40	0.97 ± 0.023	0.32 ± 0.026	0.53 ± 0.015	1.05 ± 0.023	1.20 ± 0.032	0.61 ± 0.017	0.65 ± 0.020	0.13 ± 0.017	0.63 ± 0.023	1.07 ± 0.026	1.47 ± 0.026	2.14 ± 0.020	1.21 ± 0.026	0.75 ± 0.026	0.75 ± 0.032	0.39 ± 0.026
CGM20J	0.94 ± 0.029	0.34 ± 0.020	0.54 ± 0.017	1.10 ± 0.029	1.19 ± 0.032	0.62 ± 0.023	0.65 ± 0.023	0.11 ± 0.020	0.67 ± 0.020	1.11 ± 0.032	1.38 ± 0.020	2.13 ± 0.023	1.22 ± 0.026	0.77 ± 0.023	0.70 ± 0.020	0.42 ± 0.026
CGM40J	0.90 ± 0.032	0.33 ± 0.023	0.52 ± 0.020	1.12 ± 0.020	1.17 ± 0.032	0.62 ± 0.029	0.63 ± 0.026	0.12 ± 0.020	0.62 ± 0.020	1.11 ± 0.017	1.39 ± 0.026	2.16 ± 0.032	1.19 ± 0.023	0.81 ± 0.023	0.71 ± 0.020	0.43 ± 0.017
SPC20J	0.96 ± 0.020	0.36 ± 0.020	0.56 ± 0.026	1.05 ± 0.015	1.20 ± 0.032	0.57 ± 0.032	0.66 ± 0.023	0.11 ± 0.017	0.64 ± 0.020	1.08 ± 0.032	1.43 ± 0.026	2.10 ± 0.023	1.26 ± 0.026	0.74 ± 0.020	0.73 ± 0.020	0.40 ± 0.017
SPC40J	0.97 ± 0.026	0.34 ± 0.020	0.51 ± 0.026	1.04 ± 0.026	1.19 ± 0.032	0.59 ± 0.029	0.65 ± 0.032	0.13 ± 0.020	0.62 ± 0.026	1.06 ± 0.020	1.45 ± 0.026	2.11 ± 0.032	1.23 ± 0.026	0.76 ± 0.020	0.74 ± 0.023	0.38 ± 0.020
*p*‐value	*p* > 0.5	*p* > 0.8	*p* > 0.6	*p* > 0.1	*p* > 0.7	*p* > 0.8	*p* > 0.7	*p* > 0.9	*p* > 0.6	*p* > 0.5	*p* > 0.2	*p* > 0.6	*p* > 0.5	*p* > 0.5	*p* > 0.7	*p* > 0.2
Main effect: replacement source																
CGM	0.92 ± 0.017^B^	0.33 ± 0.014	0.54 ± 0.011	1.11 ± 0.012^A^	1.18 ± 0.016	0.62 ± 0.015	0.64 ± 0.016	0.11 ± 0.009	0.64 ± 0.014	1.12 ± 0.015^A^	1.39 ± 0.014^B^	2.15 ± 0.015	1.21 ± 0.013	0.78 ± 0.015	0.71 ± 0.011	0.44 ± 0.014^A^
SPC	0.96 ± 0.013^A^	0.34 ± 0.013	0.54 ± 0.016	1.05 ± 0.011^B^	1.20 ± 0.016	0.59 ± 0.014	0.66 ± 0.013	0.12 ± 0.011	0.64 ± 0.013	1.08 ± 0.015^B^	1.45 ± 0.016^A^	2.12 ± 0.014	1.24 ± 0.015	0.75 ± 0.013	0.74 ± 0.014	0.39 ± 0.012^B^
Main effect: replacement level																
20%	0.94 ± 0.014	0.35 ± 0.012	0.56 ± 0.014^A^	1.08 ± 0.013	1.20 ± 0.016	0.60 ± 0.016	0.66 ± 0.013	0.11 ± 0.010	0.66 ± 0.012	1.10 ± 0.017	1.42 ± 0.017	2.13 ± 0.013	1.23 ± 0.014	0.75 ± 0.014	0.72 ± 0.012	0.42 ± 0.016
40%	0.94 ± 0.019	0.32 ± 0.014	0.52 ± 0.011^B^	1.08 ± 0.017	1.18 ± 0.016	0.61 ± 0.015	0.64 ± 0.015	0.12 ± 0.010	0.63 ± 0.013	1.10 ± 0.018	1.43 ± 0.019	2.14 ± 0.016	1.21 ± 0.014	0.78 ± 0.015	0.73 ± 0.014	0.41 ± 0.015
Main effect: jack mackerel meal																
Without	0.94 ± 0.016	0.33 ± 0.014	0.55 ± 0.013	1.08 ± 0.013	1.19 ± 0.016	0.61 ± 0.014	0.65 ± 0.015	0.12 ± 0.010	0.65 ± 0.014	1.11 ± 0.018	1.43 ± 0.019	2.14 ± 0.013	1.22 ± 0.013	0.76 ± 0.016	0.73 ± 0.014	0.42 ± 0.018
With	0.94 ± 0.018	0.34 ± 0.012	0.53 ± 0.014	1.08 ± 0.017	1.19 ± 0.017	0.60 ± 0.017	0.65 ± 0.014	0.12 ± 0.011	0.64 ± 0.013	1.09 ± 0.015	1.41 ± 0.016	2.13 ± 0.016	1.23 ± 0.016	0.77 ± 0.014	0.72 ± 0.012	0.41 ± 0.013
Three‐way ANOVA																
Replacement source	*p* < 0.05	*p* > 0.4	*p* > 0.6	*p* < 0.002	*p* > 0.3	*p* > 0.1	*p* > 0.2	*p* > 0.3	*p* > 0.8	*p* < 0.05	*p* < 0.006	*p* > 0.1	*p* > 0.08	*p* > 0.08	*p* > 0.1	*p* < 0.02
Replacement level	*p* > 0.8	*p* > 0.2	*p* < 0.05	*p* > 0.9	*p* > 0.3	*p* > 0.5	*p* > 0.2	*p* > 0.4	*p* > 0.09	*p* > 0.9	*p* > 0.6	*p* > 0.3	*p* > 0.2	*p* > 0.1	*p* > 0.4	*p* > 0.5
Jack mackerel meal	*p* > 0.5	*p* > 0.9	*p* > 0.3	*p* > 0.3	*p* > 0.8	*p* > 0.8	*p* > 0.5	*p* > 0.8	*p* > 0.9	*p* > 0.5	*p* > 0.4	*p* > 0.3	*p* > 0.3	*p* > 0.6	*p* > 0.4	*p* > 0.5
Replacement source × replacement level	*p* > 0.2	*p* > 0.6	*p* > 0.6	*p* > 0.3	*p* > 0.7	*p* > 0.7	*p* > 0.5	*p* > 0.7	*p* > 0.7	*p* > 0.3	*p* > 0.5	*p* > 0.6	*p* > 0.7	*p* > 0.4	*p* > 0.7	*p* > 0.6
Replacement source × Jack mackerel meal	*p* > 0.8	*p* > 0.9	*p* > 0.9	*p* > 0.6	*p* > 0.7	*p* > 0.7	*p* > 0.9	*p* > 0.6	*p* > 0.6	*p* > 0.7	*p* > 0.6	*p* > 0.5	*p* > 0.4	*p* > 0.8	*p* > 0.6	*p* > 0.4
Replacement level × Jack mackerel meal	*p* > 0.6	*p* > 0.9	*p* > 0.9	*p* > 0.8	*p* > 0.7	*p* > 0.8	*p* > 0.6	*p* > 0.7	*p* > 0.8	*p* > 0.7	*p* > 0.8	*p* > 0.8	*p* > 0.7	*p* > 0.7	*p* > 0.6	*p* > 0.6
Replacement source × replacement level × Jack mackerel meal	*p* > 0.7	*p* > 0.9	*p* > 0.9	*p* > 0.9	*p* > 0.9	*p* > 0.9	*p* > 0.8	*p* > 0.9	*p* > 0.4	*p* > 0.7	*p* > 0.8	*p* > 0.8	*p* > 0.8	*p* > 0.8	*p* > 0.8	*p* > 0.6

*Note:* Values (means of triplicates ± SE) with different uppercase letters indicate significant differences (*p* < 0.05) based on three‐way ANOVA.

Abbreviations: Ala, alanine; Arg, arginine; Asp, aspartic acid; Glu, glutamic acid; Gly, glycine; His, histidine; Ile, isoleucine; Leu, leucine; Lys, lysine; Phe, phenylalanine; Pro, proline; Ser, serine; Thr, threonine; Trp, tryptophan; Tyr, tyrosine; Val, valine.

### 3.9. The FA Profiles of the Whole‐Body Red Sea Bream

The FA profiles of the whole‐body fish were clearly classified into three clusters [cluster 1 (the Con diet), cluster 2 (the CGM20, CGM40, CGM20J, and CGM40J diets), and cluster 3 (the SPC20, SPC40, SPC20J, and SPC40J diets)] (Figure 2A). The 69% variation in the PCA model was attributed to the first and second principal components (PC1 and PC2), accounting for 43% and 26%, respectively. The myristic acid (C14:0), palmitoleic acid (C16:1n−7), ∑n−3 HUFA, EPA, eicosenoic acid (C20:1n−9), tetracosenoic acid (C24:1n−9), ∑MUFA, docosadienoic acid (C22:2n−6), and DHA were determined to be the most significant variables in PC1, and the most statistical variables in PC2 were ∑SFA, palmitic acid (C16:0), and stearic acid (C18:0) (Figure 2B). Furthermore, ∑SFA, ∑MUFA, ∑n‐3 HUFA, EPA, DHA, docosadienoic acid, eicosenoic acid, magaoleic acid (C17:1n‐7), myristic acid, palmitoleic acid, and tetracosenoic acid were strongly correlated with the FA profiles of the whole‐body fish fed the Con diet, while oleic acid (C18:1n−9), *γ*‐linolenic acid (C18:3n−6), linoleic acid (C18:2n−6), and palmitic acid, stearic acid, erucic acid (C22:1n−9), and linolenic acid (C18:3n−3) were highly correlated with the FA profiles of the whole‐body fish fed the CGM‐ and SPC‐replaced diets, respectively.

Figure 2Principal component analysis score plot (A) and correlation loading plot (B) for the fatty acid profiles of the whole‐body red sea bream fed the experimental diets. ∑SFA: total saturated fatty acids; ∑MUFA: total monounsaturated fatty acids; ∑n−3 HUFA: total n−3 highly unsaturated fatty acids.

**Figure:**
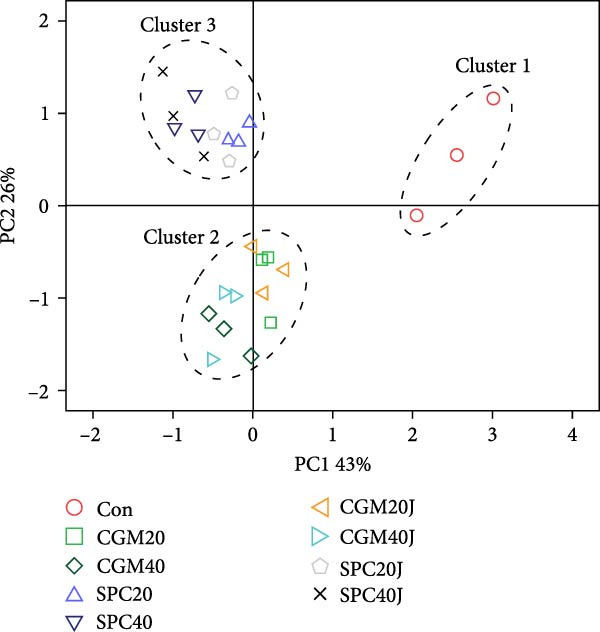
(A)

**Figure:**
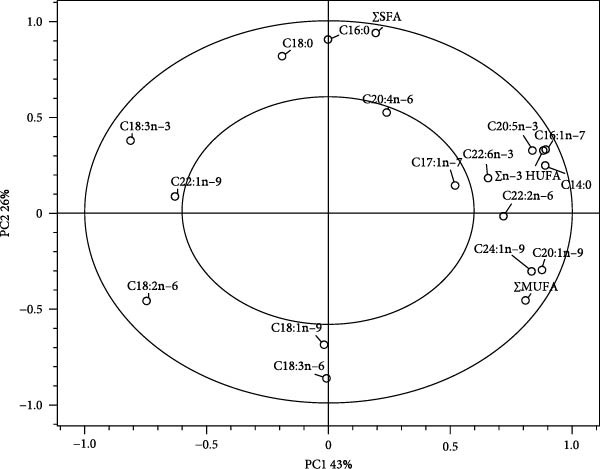
(B)

The SPC‐replaced diets (21.37% of total FA) attained significantly (*p* < 0.0001) higher ∑SFA of the whole‐body fish than the CGM‐replaced diets (20.30% of total FA) (Table 10). Furthermore, dietary replacements of 20% FM (20.94% of total FA) attained significantly (*p* < 0.02) higher the ∑SFA of the whole‐body fish than those of 40% FM (20.74% of total FA). However, dietary JMM inclusion did not significantly (*p* > 0.8) affect the ∑SFA of the whole‐body fish. The CGM‐replaced diets (32.56% of total FA) attained significantly (*p* < 0.0001) higher ∑MUFA of the whole‐body fish than the SPC‐replaced diets (31.85% of total FA). However, dietary replacement level and JMM inclusion did not significantly (*p* > 0.08 and *p* > 0.1, respectively) impact the ∑MUFA of the whole‐body fish. The SPC‐replaced diets (6.93% of total FA) attained significantly (*p* < 0.003) higher ∑n‐3 HUFA of the whole‐body fish than the CGM‐replaced diets (6.76% of total FA). Dietary replacements of 20% FM (6.95% of total FA) achieved significantly (*p* < 0.0001) higher ∑n‐3 HUFA of the whole‐body fish than those of 40% FM (6.74% of total FA). Furthermore, the all plant‐replaced diets without JMM inclusion (6.97% of total FA) attained significantly (*p* < 0.0001) higher ∑n‐3 HUFA of the whole‐body fish than those with JMM inclusion (6.72% of total FA). In the multiple comparison test, the ∑SFA of the whole‐body fish fed the Con, SPC20, SPC40, SPC20J, and SPC40J diets was significantly (*p* < 0.0001) higher than that of fish fed the CGM20, CGM40, CGM20J, and CGM40J diets. The ∑MUFA of the whole‐body fish fed the Con diet was significantly (*p* < 0.0001) higher than that of fish fed the SPC20, SPC40, CGM20J, CGM40J, SPC20J, and SPC40J diets. The highest ∑n−3 HUFA (8.39% of total FA) was found in fish fed the Con diet.

**Table 10:** The whole‐body fatty acid (% of total fatty acids) profiles of red sea bream fed the experimental diets for 8 weeks.

Experimental diets	C14:0	C16:0	C18:0	∑SFA ^1^	C16:1n‐7	C17:1n‐7	C18:1n‐9	C20:1n‐9	C22:1n‐9	C24:1n‐9	∑MUFA^2^	C18:2n–6	C18:3n–3	C18:3n–6	C20:4n–6	C20:5n–3	C22:2n–6	C22:6n–3	∑n–3 HUFA^3^	Unknown
Con	2.12 ± 0.049^a^	14.24 ± 0.066^ab^	5.19 ± 0.069^ab^	21.54 ± 0.052^a^	3.38 ± 0.090^a^	0.41 ± 0.026	24.91 ± 0.309	2.74 ± 0.061^a^	0.36 ± 0.026	1.69 ± 0.038^a^	33.49 ± 0.121^a^	28.17 ± 0.133^e^	2.96 ± 0.043^d^	0.56 ± 0.038^ab^	1.74 ± 0.090	4.36 ± 0.029^a^	0.41 ± 0.038	4.03 ± 0.061^a^	8.39 ± 0.032^a^	2.75 ± 0.188
CGM20	1.67 ± 0.069^b^	13.78 ± 0.058^bc^	5.08 ± 0.043^ab^	20.53 ± 0.084^b^	2.83 ± 0.066^b^	0.37 ± 0.026	25.02 ± 0.243	2.47 ± 0.075^b^	0.40 ± 0.032	1.40 ± 0.032^bc^	32.49 ± 0.232^ab^	32.25 ± 0.153^b^	3.06 ± 0.043^cd^	0.65 ± 0.043^ab^	1.67 ± 0.038	3.20 ± 0.040^cd^	0.35 ± 0.020	3.75 ± 0.038^b^	6.95 ± 0.078^bcd^	2.05 ± 0.554
CGM40	1.64 ± 0.035^b^	13.47 ± 0.078^c^	4.97 ± 0.087^b^	20.08 ± 0.044^b^	2.74 ± 0.029^b^	0.37 ± 0.017	25.20 ± 0.205	2.35 ± 0.029^bc^	0.42 ± 0.018	1.38 ± 0.023^bc^	32.46 ± 0.232^ab^	33.03 ± 0.176^a^	3.17 ± 0.038^c^	0.70 ± 0.032^a^	1.65 ± 0.032	3.11 ± 0.038^de^	0.30 ± 0.020	3.64 ± 0.035^b^	6.75 ± 0.003^cde^	1.85 ± 0.350
SPC20	1.71 ± 0.012^b^	14.37 ± 0.107^a^	5.31 ± 0.069^a^	21.39 ± 0.082^a^	2.84 ± 0.038^b^	0.36 ± 0.017	24.84 ± 0.088	2.28 ± 0.026^bcd^	0.41 ± 0.020	1.21 ± 0.040^cd^	31.94 ± 0.126^bcd^	31.50 ± 0.107^bcd^	3.24 ± 0.055^bc^	0.55 ± 0.032^ab^	1.73 ± 0.035	3.43 ± 0.035^b^	0.33 ± 0.026	3.77 ± 0.049^b^	7.20 ± 0.015^b^	2.12 ± 0.265
SPC40	1.64 ± 0.030^b^	14.41 ± 0.061^a^	5.33 ± 0.093^a^	21.38 ± 0.056^a^	2.77 ± 0.021^b^	0.36 ± 0.020	24.65 ± 0.168	2.17 ± 0.023^cde^	0.47 ± 0.032	1.18 ± 0.043^d^	31.60 ± 0.173^d^	31.97 ± 0.124^b^	3.38 ± 0.043^ab^	0.54 ± 0.026^ab^	1.69 ± 0.038	3.33 ± 0.032^bc^	0.30 ± 0.017	3.66 ± 0.059^b^	7.00 ± 0.090^bc^	2.15 ± 0.423
CGM20J	1.62 ± 0.032^b^	13.70 ± 0.088^c^	5.14 ± 0.035^ab^	20.46 ± 0.086^b^	2.83 ± 0.042^b^	0.36 ± 0.023	25.14 ± 0.179	2.41 ± 0.020^b^	0.43 ± 0.032	1.51 ± 0.023^ab^	32.68 ± 0.172^b^	30.89 ± 0.147^cd^	3.21 ± 0.026^bc^	0.62 ± 0.032^ab^	1.68 ± 0.038	2.95 ± 0.081^ef^	0.37 ± 0.032	3.85 ± 0.026^ab^	6.81 ± 0.090^cde^	3.28 ± 0.072
CGM40J	1.60 ± 0.024^b^	13.45 ± 0.221^c^	5.09 ± 0.049^ab^	20.14 ± 0.204^b^	2.75 ± 0.037^b^	0.34 ± 0.020	25.36 ± 0.204	2.30 ± 0.055^bcd^	0.45 ± 0.035	1.38 ± 0.049^bc^	32.59 ± 0.068^b^	31.64 ± 0.118^bc^	3.25 ± 0.032^abc^	0.68 ± 0.023^ab^	1.65 ± 0.026	2.79 ± 0.049^f^	0.33 ± 0.015	3.76 ± 0.066^b^	6.55 ± 0.062^e^	3.15 ± 0.248
SPC20J	1.67 ± 0.033^b^	14.34 ± 0.082^a^	5.36 ± 0.042^a^	21.37 ± 0.066^a^	2.87 ± 0.053^b^	0.36 ± 0.010	25.00 ± 0.084	2.13 ± 0.029^de^	0.43 ± 0.032	1.34 ± 0.049^bcd^	32.12 ± 0.025^bcd^	30.82 ± 0.245^d^	3.39 ± 0.038^ab^	0.53 ± 0.020^ab^	1.75 ± 0.032	2.99 ± 0.038^def^	0.33 ± 0.020	3.86 ± 0.060^ab^	6.84 ± 0.077^cde^	2.85 ± 0.097
SPC40J	1.60 ± 0.032^b^	14.37 ± 0.101^a^	5.38 ± 0.073^a^	21.35 ± 0.129^a^	2.77 ± 0.047^b^	0.35 ± 0.021	24.89 ± 0.163	2.05 ± 0.040^e^	0.48 ± 0.015	1.22 ± 0.055^cd^	31.76 ± 0.113^cd^	30.89 ± 0.165^cd^	3.45 ± 0.042^a^	0.51 ± 0.046^b^	1.72 ± 0.038	2.90 ± 0.035^ef^	0.32 ± 0.020	3.77 ± 0.058^ab^	6.67 ± 0.047^de^	3.34 ± 0.170
*p*‐value	*p* < 0.0001	*p* < 0.0001	*p* < 0.002	*p* < 0.0001	*p* < 0.0001	*p* > 0.5	*p* > 0.3	*p* < 0.0001	*p* > 0.2	*p* < 0.0001	*p* < 0.0001	*p* < 0.0001	*p* < 0.0001	*p* < 0.005	*p* > 0.6	*p* < 0.0001	*p* > 0.1	*p* < 0.002	*p* < 0.0001	—
Main effect: replacement source																				
CGM	1.63 ± 0.025	13.60 ± 0.085^B^	5.07 ± 0.037^B^	20.30 ± 0.095^B^	2.79 ± 0.029	0.36 ± 0.012	25.18 ± 0.118^A^	2.38 ± 0.035^A^	0.43 ± 0.017	1.42 ± 0.026^A^	32.56 ± 0.103^A^	31.95 ± 0.301^A^	3.17 ± 0.032^B^	0.66 ± 0.020^A^	1.66 ± 0.018^B^	3.01 ± 0.065^B^	0.34 ± 0.015	3.75 ± 0.036	6.76 ± 0.064^B^	—
SPC	1.65 ± 0.021	14.37 ± 0.048^A^	5.34 ± 0.039^A^	21.37 ± 0.046^A^	2.81 ± 0.027	0.36 ± 0.009	24.84 ± 0.083^B^	2.16 ± 0.034^B^	0.45 ± 0.017	1.24 ± 0.033^B^	31.85 ± 0.096^B^	31.29 ± 0.195^B^	3.36 ± 0.036^A^	0.54 ± 0.018^B^	1.72 ± 0.021^A^	3.16 ± 0.085^A^	0.32 ± 0.012	3.77 ± 0.039	6.93 ± 0.079^A^	—
Main effect: replacement level																				
20%	1.67 ± 0.025	14.05 ± 0.122	5.22 ± 0.050	20.94 ± 0.169^A^	2.84 ± 0.027^A^	0.36 ± 0.011	25.00 ± 0.094	2.32 ± 0.054^A^	0.42 ± 0.016	1.36 ± 0.044^A^	32.31 ± 0.136	31.36 ± 0.230^B^	3.23 ± 0.048^B^	0.59 ± 0.025	1.71 ± 0.023	3.14 ± 0.076^A^	0.34 ± 0.014	3.81 ± 0.029^A^	6.95 ± 0.067^A^	—
40%	1.62 ± 0.017	13.93 ± 0.185	5.19 ± 0.074	20.74 ± 0.241^B^	2.76 ± 0.019^B^	0.36 ± 0.011	25.03 ± 0.140	2.22 ± 0.048^B^	0.45 ± 0.016	1.29 ± 0.042^B^	32.10 ± 0.180	31.88 ± 0.294^A^	3.31 ± 0.044^A^	0.61 ± 0.035	1.68 ± 0.020	3.03 ± 0.080^B^	0.31 ± 0.011	3.71 ± 0.036^B^	6.74 ± 0.068^B^	—
Main effect: jack mackerel meal																				
Without	1.66 ± 0.025	14.01 ± 0.152	5.17 ± 0.069	20.84 ± 0.211	2.80 ± 0.027	0.37 ± 0.011	24.93 ± 0.123	2.32 ± 0.046^A^	0.43 ± 0.016	1.29 ± 0.041^B^	32.12 ± 0.172	32.19 ± 0.219^A^	3.21 ± 0.048^B^	0.61 ± 0.030	1.68 ± 0.021	3.27 ± 0.049^A^	0.32 ± 0.013	3.71 ± 0.032^B^	6.97 ± 0.067^A^	—
With	1.62 ± 0.019	13.97 ± 0.164	5.24 ± 0.055	20.83 ± 0.211	2.81 ± 0.029	0.35 ± 0.010	25.10 ± 0.107	2.22 ± 0.056^B^	0.45 ± 0.017	1.36 ± 0.046^A^	32.29 ± 0.149	31.06 ± 0.155^B^	3.32 ± 0.040^A^	0.59 ± 0.030	1.70 ± 0.022	2.91 ± 0.040^B^	0.34 ± 0.014	3.81 ± 0.033^A^	6.72 ± 0.057^B^	—
Three‐way ANOVA																				
Replacement source	*p* > 0.4	*p* < 0.0001	*p* < 0.0001	*p* < 0.0001	*p* > 0.4	*p* > 0.9	*p* < 0.02	*p* < 0.0001	*p* > 0.3	*p* < 0.0001	*p* < 0.0001	*p* < 0.0001	*p* < 0.0001	*p* < 0.0001	*p* < 0.04	*p* < 0.0001	*p* > 0.2	*p* > 0.7	*p* < 0.003	—
Replacement level	*p* > 0.08	*p* > 0.1	*p* > 0.5	*p* < 0.02	*p* < 0.02	*p* > 0.7	*p* > 0.8	*p* < 0.03	*p* > 0.08	*p* < 0.03	*p* > 0.08	*p* < 0.0001	*p* < 0.008	*p* > 0.4	*p* > 0.2	*p* < 0.004	*p* > 0.07	*p* < 0.02	*p* < 0.0001	—
Jack mackerel meal	*p* > 0.1	*p* > 0.6	*p* > 0.1	*p* > 0.8	*p* > 0.7	*p* > 0.4	*p* > 0.1	*p* < 0.006	*p* > 0.3	*p* < 0.03	*p* > 0.1	*p* < 0.0001	*p* < 0.002	*p* > 0.2	*p* > 0.4	*p* < 0.0001	*p* > 0.3	*p* < 0.009	*p* < 0.0001	—
Replacement source × Replacement level	*p* > 0.3	*p* > 0.05	*p* > 0.2	*p* < 0.03	*p* > 0.9	*p* > 0.9	*p* > 0.1	*p* > 0.7	*p* > 0.4	*p* > 0.9	*p* > 0.2	*p* < 0.05	*p* > 0.7	*p* > 0.1	*p* > 0.7	*p* > 0.5	*p* > 0.5	*p* > 0.9	*p* > 0.6	—
Replacement source × Jack mackerel meal	*p* > 0.9	*p* > 0.9	*p* > 0.6	*p* > 0.8	*p* > 0.8	*p* > 0.7	*p* > 0.8	*p* > 0.1	*p* > 0.7	*p* > 0.6	*p* > 0.9	*p* < 0.05	*p* > 0.9	*p* > 0.9	*p* > 0.7	*p* < 0.03	*p* > 0.5	*p* > 0.8	*p* > 0.09	—
Replacement level × Jack mackerel meal	*p* > 0.8	*p* > 0.9	*p* > 0.7	*p* > 0.7	*p* > 0.9	*p* > 0.5	*p* > 0.7	*p* > 0.7	*p* > 0.9	*p* > 0.1	*p* > 0.8	*p* > 0.3	*p* > 0.2	*p* > 0.9	*p* > 0.9	*p* > 0.6	*p* > 0.5	*p* > 0.7	*p* > 0.9	—
Replacement source × Replacement level × Jack mackerel meal	*p* > 0.8	*p* > 0.8	*p* > 0.7	*p* > 0.6	*p* > 0.7	*p* > 0.9	*p* > 0.9	*p* > 0.8	*p* > 0.9	*p* > 0.8	*p* > 0.9	*p* > 0.4	*p* > 0.9	*p* > 0.7	*p* > 0.8	*p* > 0.5	*p* > 0.8	*p* > 0.9	*p* > 0.6	—

*Note:* Values (means of triplicates ± SE) with different lowercase and uppercase letters indicate significant differences (*p* < 0.05) based on Tukey’s honestly significant differences test and three‐way ANOVA, respectively.

^1^∑SFA, total saturated fatty acids.

^2^∑MUFA, total monounsaturated fatty acids.

^3^∑n–3 HUFA, total n–3 highly unsaturated fatty acids.

## 4. Discussion

The effectiveness of FM substitutes in fish diets may vary depending on the quality of the replacement and the level of substitution [64, 65]. The superior growth performance (WG and SGR) and FC observed in red sea bream fed the CGM‐replaced diets compared to the SPC‐replaced diets indicate that CGM is a more efficient FM replacer than SPC in the red sea bream diet in this study. Likewise, growth performance and FC of rockfish fed the CGM‐replaced diets were superior to fish fed the corn protein concentrate (CPC)‐ and SPC‐replaced diets when juvenile rockfish were supplied with a 55% FM‐based diet or diets substituting 25% and 50% of FM with CGM, CPC, and SPC with 22% JMM inclusion [66]. Similarly, Baek et al. [67] revealed that slightly, but not significantly, greater growth performance and FC of fish fed the CGM‐substituted diets compared to fish fed the SPC‐ and CPC‐substituted diets were found when juvenile olive flounder were supplied with a 60% FM‐based diet or diets substituting 25% and 50% of FM by CGM, CPC, and SPC with 12% JMM inclusion.

Dietary replacements of 20% FM resulted in better growth performance and FC compared to those of 40% FM. This might be explained by the fact that higher FM substitution with plant proteins in diets led to poorer palatability, resulting in impaired FC and growth performance of carnivorous fish, such as turbot (*Psetta maxima*) and orange‐spotted grouper (*Epinephelus coioides*) [46, 68]. Likewise, increased dietary replacement levels with fermented rapeseed meal for FM decreased FC and growth performance of red sea bream when fish were provided with a 47% FM‐based diet or diets replacing 18.7%, 37.5%, 56.3%, and 75.0% of FM by fermented rapeseed meal for 65 days [69]. Furthermore, SGR and FC of silvery‐black porgy (*Sparidentex hasta*) linearly decreased with increased (15%–75%) FM substitution levels with combined isolated soy protein and soybean meal in a 62% FM‐based diet [47]. Therefore, the significant decrease in the growth performance and FC of red sea bream at higher FM substitution levels in this study supports the growth performance benefits observed at dietary 20% substitution of FM.

Substitutability of FM with a replacer in fish diets can be also highly influenced by inclusion of feed attractant and result in improvement in FC [51, 52]. The all‐plant‐replaced diets with JMM inclusion produced higher FC and growth performance of red sea bream compared to those without JMM inclusion, indicating that JMM had an improvement effect on FC in red sea bream in the low‐FM diets. The higher His content in the all‐plant‐replaced diets with JMM inclusion compared to those without JMM inclusion resulted in superior WG and SGR in red sea bream. This is likely due to higher FC in former diets. Likewise, His in the muscle extracts of jack mackerel was identified as an effective feed stimulant for olive flounder and Pacific bluefin tuna (*Thunnus orientalis*) [70, 71]. Similarly, FM up to 18.2% could be substituted with CGM without compromising WG and daily FC of rockfish [72], on the other hand, 25% of FM could be replaced by CGM in diets with 22% JMM inclusion without any negative effect on growth and FC of rockfish [66]. Therefore, manipulation of JMM having an improvement effect on FC could increase palatability of the low‐FM diets and elevate FC of carnivorous fish. Likewise, the 74% FM‐based diet of olive flounder led to superior SGR compared to all other low‐FM diets when juvenile fish were supplied with a 74% FM‐based diet or one of diets replacing various (25%–100%) levels of FM with SPC or 75% of FM with SPC supplemented with AA [73]. However, unlike Deng et al. [73], 25% of FM could be replaced by SPC and CGM with 12% JMM inclusion in diets without lowering WG and FC of olive flounder [67]. Similarly, Tharaka et al. [74] also proved that 50% FM replacement by the blended proteins in diet with inclusion of 6% Antarctic krill (*Euphausia superba*) meal produced superior growth of olive flounder compared to a 56% FM‐based diet when juvenile fish were provided with a 56% FM‐based diet or diets replacing 50% FM by the blended of SPC, wheat gluten, tankage meal, and poultry by‐product meal with inclusion of 3%, 6%, 9%, and 12% Antarctic krill meal. These findings support the conclusion that feed ingredient exhibiting strong feed attractant activity, such as JMM, can effectively mitigate the negative effects of FM decrease.

Comparable WG, SGR, and FC of red sea bream fed the Con diet to fish fed the CGM20, CGM20J, and SPC20J diets suggested that FM up to 20% could be substitutable by CGM with or without JMM inclusion or SPC with JMM inclusion in the 60% FM‐based diet without significantly worsening growth and FC. Additionally, FC of red sea bream was directly reflected in growth performance in this experiment. Similarly, CGM could replace FM up to 14.8% and 28.6% in the diet of puffer and Asian seabass (*Lates calcarifer*), respectively, without compromising WG and FC [29, 34].

However, supplementation of likely deficient AA in CGM into the low‐FM diets could improve the substitutability of FM in fish diets. FM up to 40% could be substitutable by CGM supplemented with AA (Arg, Lys, and Trp) in the 75% FM‐based diet without deteriorating growth of olive flounder, whereas 40% FM replacement by CGM without AA supplementation in diet led to poor growth [35]. Furthermore, FM up to 60% could be substituted by CGM in diet with AA supplementation, causing no reduction in growth performance and FC of spotted rose snapper when juvenile fish were supplied with a 54.8% FM‐based diet or one of diets replacing 20% and 40% of FM by CGM with Arg supplementation or 60%, 80%, and 100% of FM by CGM with Arg and Lys supplementation [33]. As Arg and Lys are the limiting AA in CGM [29], dietary elevated FM replacements with CGM led to decreased all EAA including Arg and Lys, except for increased Leu and Phe in the present experiment. Therefore, reduced EAA resulted from dietary increased FM replacements with CGM might be one of the reasons why the CGM40 diet lowered growth performance of fish. Nevertheless, as supplementation of the synthetic AA does not always improve dietary substitutability of FM, the use of the synthetic AA in fish diets still remains a contentious issue [75, 76]. Rather than relying solely on the synthetic AA, the use of blended protein sources can be an effective strategy to increase the substitutability of FM in fish feeds, as it may improve the overall nutritional quality by compensating for the deficiencies or imbalances of single ingredient [77, 78]. Moreover, using blended protein sources in fish feeds is often more economical and practical, enabling higher levels of FM replacement compared to single‐source alternative [79]. Therefore, future study should explore the potential of blended protein sources including CGM, which proved to be a more efficient FM replacer than SPC in this study, in the red sea bream diets to improve dietary FM replacement.

Inferior growth performance and FC of fish fed the SPC20 diet compared to fish fed the Con diet indicated that SPC could not replace 20% of FM in the 60% FM‐based diet of juvenile fish. Likewise, dietary 25% FM substitution by SPC led to poorer growth of juvenile (IW of ca. 2.5 g) olive flounder compared to fish fed the 74% FM‐based diet [73]. Unlike this study, however, SPC could replace 50% of FM in the 60% FM‐based diet for fingerling (IW of 50.2 g) red sea bream without deteriorating growth and FC [36]. The substitutability of FM with SPC in diets may vary depending on the size (or age) of fish, as smaller fish are likely to replacer less FM with a substitute compared to larger fish. For instance, 6% and 24% FM protein could be substituted by FSM in the diets of juvenile (IW of 1.2 g) and grower (IW of 148.2 g) rockfish, respectively, without harming growth and FC [45]. Additionally, 30% and 70% of FM could be replaceable by CGM in the diets of fingerling (IW of 53 g) and grower (IW of 280 g) red sea bream, respectively, without lowering growth when fingerling and grower fish were supplied with a 50% FM‐based diet or diets substituting 30%, 50%, 70%, 90%, and 100% of FM with CGM for 40 and 232 days, respectively [44]. Therefore, it is important to consider fish size when determining the replacement level of FM in fish diets, as larger fish tend to tolerate higher levels of FM substitution.

The attractiveness of experimental diets to red sea bream was assessed through feeding behavior analysis, showing strong responses within the first 10 min. A water‐soluble extract of each diet stimulated the olfactory senses of fish, eventually triggering their feeding behavior. This was similar to the previously reported feeding behaviors of olive flounder [55, 57], rockfish [56, 59], and red sea bream [54]. Therefore, the results of this experiment could be considered a reliable and practical approach to assess the attractiveness of the experimental diets to red sea bream. The feeding behavior of fish is affected by a variety of sensory systems, including vision, olfaction, acoustic, and gustation; the olfaction system decides the attractiveness of food, whereas the gustation system performs the final assessment of the sensory and nutritional qualities of food, ultimately influencing the actual consumption of fish [80]. The attractiveness of the Con, SPC20J, and CGM20J diets to fish was the highest in the 1st, 2nd, and 3rd tests, respectively, at the end of the 30‐min test (Table 4). No significant differences in the attractiveness of the Con diet compared to the CGM20J diet, but higher than that of the SPC20J diet in the 4th test indicated that the attractiveness of the Con diet to fish was similar to that of the CGM20J diet. This might explain why the CGM20J diet brought about similar FC compared to the Con diet in the 8‐week practical feeding experiment. Furthermore, His, and L‐His, L‐Glu, and inosine monophosphate from the muscle extracts of jack mackerel were considered the highly effective feed stimulants for olive flounder and Pacific bluefin tuna, respectively [70, 71].

Dietary requirements of Arg, Lys, and Val for red sea bream were reported to be 2.37%, 1.79%, and 0.90%, respectively [6, 8, 9], and all experimental diets appeared to fulfill their requirements. However, inferior growth performance of red sea bream fed the SPC20, SPC40, and SPC40J diets compared to fish fed the Con diet might be attributed to the high Arg content in the SPC‐replaced diet, except for the SPC20J diet. The Arg is a limiting AA for growth and its deficiency in diet could cause reduced growth, but excessive dietary Arg could also lead to a negative effect on growth of juvenile cobia (*Rachycentron canadum*) and yellow grouper (*Epinephelus awoara*) [81, 82]. Additionally, Rahimnejad and Lee [9] demonstrated that WG and SGR of red sea bream fed a diet containing 2.54% Arg were the greatest, but further increased dietary Arg content led to a slight, but not significant decrease in growth performance when red sea bream were provided with the diets containing various (1.42%, 1.88%, 2.22%, 2.54%, 3.08%, and 3.43%) levels of Arg. The Arg in SPC was higher than that of all other main proteins (FM, JMM, and CGM) and it was well reflected in all experimental diets. All SPC‐replaced diets, except for the SPC20J diet, contained over 3.03% in Arg. Therefore, high Arg content in the SPC‐replaced diets might be one of the reasons why the growth performance of fish fed the SPC20, SPC40, and SPC40J diets was negatively affected. Similarly, WG and SGR of juvenile golden pompano (*Trachinotus ovatus*) fed a diet containing 2.65% Arg were the highest, but further increased Arg beyond 2.98% significantly reduced WG and SGR when juvenile golden pompano were provided with the diets containing 2.05%, 2.38%, 2.65%, 2.98%, 3.25%, and 3.58% Arg [83].

The n‐3 HUFA are critical for marine fish, and insufficient supply in fish feeds adversely affect growth of fish [84, 85]. The Con diet showed the highest ∑n−3 HUFA, as FM contained higher ∑n−3 HUFA compared to other alternative protein sources (CGM and SPC). The higher ∑n−3 HUFA in diets with 20% FM replacement compared to those with 40% FM replacement might be one of the reasons for the poorer growth performance of red sea bream in the former compared the latter. Likewise, the growth of black sea bream (*Acanthopagrus schlegeli*), olive flounder, and yellow croaker (*Larimichthys crocea*) tended to decrease when dietary ∑n−3 HUFA was lower than dietary optimal ∑n−3 HUFA [84–86].

Growth performance of red sea bream appeared to be proportional to FC in this experiment, which resulted in no significant differences in feed utilization. Likewise, feed utilization of black sea bream was unaffected by 20% and 40% FM substitution with CGM, pea protein isolate, and their combination in a 66% FM‐based diet [16]. Similarly, FE and PER of large yellow croaker fed a 40% FM‐based diet were comparable to fish fed all SPC‐substituted diets when large yellow croaker were supplied with a 40% FM‐based diet or diets substituting 15%, 30%, and 45% of FM with SPC [28]. In addition, FE and PER of olive flounder fed a 56% FM‐based diet were similar to fish fed all low‐FM diets supplemented with various levels of Antarctic krill meal [74].

CF values of less than 1 mean that fish are in poor health, while values of CF greater than 1 mean that fish are in good health [87]. Although all red sea bream in the present experiment had CF values of higher than 1, the diets with 40% FM replacements resulted in the lower CF compared to the diets with 20% FM replacements. Similarly, the highest and lowest CF values were found in olive flounder fed the low‐FM diets replacing 50% FM with the combined proteins with 6% Antarctic krill meal inclusion and without Antarctic krill meal inclusion, respectively [74]. Likewise, starry flounder fed a 68% FM‐based diet replacing 100% of FM with SPC produced the lowest CF, well reflected from the poorest growth performance [40].

VSI and HSI are the effective indicators of dietary nutrient absorption and health in fish [88]. In particular, higher VSI and HSI were observed in diets with 40% FM replacements compared to those with 20% FM replacements, likely due to higher carbohydrate content in the former. VSI and HSI of fish are closely influenced by dietary carbohydrate content, and the high‐carbohydrate diets resulted in high VSI and HSI because of high fat and liver glycogen deposition in grass carp (*Ctenopharyngodon idella*) and golden pompano (*Trachinotus ovatus*) [89, 90]. Xu et al. [91] also unveiled that increased carbohydrate content in diets tended to increase VSI and HSI of hybrid snakehead (*Channa maculate* × *C. argus*) when fish were provided with diets including graded (5%–25%) levels of *α*‐starch. Likewise, dietary elevated carbohydrate content linearly increased VSI and HSI of juvenile cobia [92]. The poorest growth performance of red sea bream fed the SPC40 diet led to the highest VSI and HSI, suggesting that 40% FM replacement with SPC in the diet of juvenile red sea bream reduced growth performance, but increased VSI and HSI. Likewise, the poorest WG was observed in Senegalese sole (*Solea senegalensis*) fed a diet replacing 100% of FM with blended plant proteins, leading to the highest HSI and VSI [93]. Similarly, silvery‐black porgy fed a diet substituting 75% of FM with the soy products (soybean meal and isolated soy protein) led to the highest HSI, but the poorest growth performance [47].

Blood parameters are able to provide an accurate assessment of health status and metabolism of fish [94]. LYZ, one of the most important antimicrobial enzymes, plays a crucial role in the innate immune system of fish [95]. SOD protects mitochondria from oxidative damage in living organisms [96]. None of blood parameters of fish were affected by dietary treatments in the current experiment, probably indicating that CGM and SPC could be substituted for FM up to 40% with or without JMM inclusion in diets without causing any undesirable impact on plasma and serum parameters of fish. Similarly, serum and plasma measurements of rockfish were not affected by either FM replacement sources (CGM, SPC, and CPC) or replacement level (25% and 50%) in diets with JMM inclusion [66]. Plasma (AST, ALT, TCO, and TPT) parameters of olive flounder fed the low‐FM diets substituting 50% of FM with blended animal and plant proteins were not influenced by the inclusion of various levels of Antarctic krill, but were significantly serum LYZ activity and SOD [74]. Additionally, serum LYZ activity of *Pseudobagrus ussuriensis* linearly decreased with dietary elevated FM replacements with CGM [32].

No significant differences in the chemical composition of the whole‐body in the current experiment might imply that dietary replacement of CGM and SPC for FM up to 40% regardless of JMM inclusion did not negatively influence the whole‐body chemical composition. Likewise, the whole‐body and dorsal muscle proximate compositions of black sea bream were not influenced by dietary 20% or 40% FM substitutions with CGM, pea protein isolate, and their combination [16]. Moreover, the chemical compositions of the flesh of largemouth bass (*Micropterus salmoides*) were unaffected by dietary FM replacements with cottonseed protein concentrate in the 12‐week feeding experiment [17]. However, dietary FM replacements with CGM, soy products (a mixture soybean meal and isolated soy protein), and SPC influenced the whole‐body proximate composition of spotted rose snapper, silvery‐black porgy, and hybrid grouper, respectively [33, 41, 47]. Therefore, dietary substitution impact of plant protein sources for FM on the whole‐body chemical composition may differ depending on the alternative plant protein sources for FM, fish species, and other experimental conditions.

Arg and Leu among EAA and Ala, Asp, and Tyr among NEAA in the whole‐body fish were significantly changed by dietary replacement source, while Ile was significantly affected by dietary replacement level in the current experiment. Nevertheless, none of EAA and NEAA in the whole‐body fish were significantly affected by dietary treatments in the multiple comparison test. No significant differences in the whole‐body AA profiles could be explained by Yamamoto et al. [97], in which the AA profiles of specific body proteins tend to remain consistent irrespective of diets, as their synthesis is determined by the genetic code in DNA. Likewise, the AA profiles of the whole‐body blunt snout bream (*Megalobrama amblycephala*) and olive flounder were unaffected by FM replacements with rice protein concentrate and diverse plant protein sources in diets, respectively [67, 98].

PCA is used to identify patterns and relationships among treatments by reducing dimensions without losing information [99]. The whole‐body FA profiles were clearly separated into 3 clusters based on the main protein sources in diets: cluster 1 (the FM‐based diet), cluster 2 (the CGM‐replaced diets), and cluster 3 (the SPC‐replaced diets). Specifically, the ∑SFA, ∑MUFA, and ∑n–3 HUFA on the right side of PC1 were strongly correlated with the FA profiles of the whole‐body fish fed the Con diet, explaining that these FA were contributors to the separation among dietary treatments. This separation was likely due to the higher content of these FA in FM than both CGM and SPC. This difference was reflected in the experimental diets and ultimately influenced the FA profiles of the whole‐body red sea bream.

The SPC‐replaced diets and all plant‐replaced diets without JMM inclusion resulted in higher ∑n–3 HUFA of the whole‐body fish than the CGM‐replaced diets and all plant‐replaced diets with JMM inclusion, respectively, while dietary replacements of 20% FM resulted in higher ∑n–3 HUFA of the whole‐body fish than those of 40% FM in the current experiment. Furthermore, higher ∑n–3 HUFA of the whole‐body was found in fish fed the Con diet compared to that of fish fed all the other diets. These findings indicated that dietary FA profiles were well reflected in the FA profiles of fish, as reported by Guerreiro et al. [15] and Ofori‐Mensah et al. [100], in which the whole‐body FA profiles well reflected from dietary FA profiles. Similarly, elevated dietary FM substitution with cottonseed protein concentrate linearly decreased EPA, which were well reflected in decreased EPA in largemouth bass [17]. Additionally, increased dietary substitution of FM with the mixture of plant proteins linearly increased ∑n–6 polyunsaturated FA (PUFA), which led to increased polyunsaturated FA in Senegalese sole [93].

This study suggests that CGM and SPC are effective protein sources for partially replacing FM (up to 20%) in red sea bream diets, regardless of JMM inclusion and with JMM inclusion, respectively. These findings indicate that CGM and SPC could serve as the viable strategies for more environmentally sustainable and economically efficient red sea bream farming. Furthermore, JMM inclusion in low‐FM diets appears to play a complementary role in enhancing growth performance and FC. Additionally, long‐term and commercial‐scale feeding trials are needed to determine the feasibility of low‐FM diets in red sea bream farm.

## 5. Conclusion

The CGM‐replaced diets achieved better growth performance and FC of red sea bream than the SPC‐replaced diets. Moreover, dietary replacements of 20% FM attained better growth performance and FC of fish than those of 40% FM. Additionally, better growth and FC was obtained in fish fed the all‐plant‐protein‐replaced diets with JMM inclusion compared to those without JMM inclusion. Growth performance and feed availability of fish fed the CGM20, CGM20J, and SPC20J diets were comparable to fish fed the Con diet. FM up to 20% could be substitutable by CGM with or without JMM inclusion and SPC with JMM inclusion in the 60% FM‐based diet of red sea bream without causing significantly negative effects on growth, feed availability, blood chemistry, and chemical composition.

## Conflicts of Interest

The authors declare no conflicts of interest.

## Author Contributions

**Yu Jin Sim**: conceptualization, investigation, data curation, writing – original draft. **Sung Hwoan Cho**: conceptualization, data curation, methodology, funding acquisition, writing – review and editing. **Tae Woong Kwon, Hae Chan Shin, Hong Min Na, Yong Woo Kwon, Seong Woo Shin and Sang Hyun Lee:** investigation, data curation. **Ki Wook Lee and Jin Choi**: funding acquisition.

## Funding

This work was supported by the National Research Foundation of Korea (NRF) grant funded by the Korean government (MSIT), 2020R1A2C1009903. This research was supported by the National Institute of Fisheries Sciences, Ministry of Oceans and Fisheries, Korea, R2026063.

## Data Availability

The data that support the findings of this study are available from the corresponding author upon reasonable request.
